# Afterhours telehealth in Australian residential aged care facilities: a mixed methods evaluation

**DOI:** 10.1186/s12913-023-10257-5

**Published:** 2023-11-15

**Authors:** Steven A. Trankle, Jennifer Reath

**Affiliations:** Department of General Practice, School of Medicine, Western Sydney University, Building 30.3.18 Campbelltown Campus, Locked Bag 1797, Penrith, NSW 2751 Australia

**Keywords:** Aged care, Telehealth, Primary healthcare, NASSS

## Abstract

**Background:**

The aged care system in Australia is under pressure. Residential aged care facilities (RACFs) and general practitioners (GPs) have difficulty providing the care needed by their residents, particularly after hours. Many residents are given ambulance support and transferred to hospital emergency departments (EDs) for care that could be provided at RACFs. The MyEmergencyDoctor (MED) service was commissioned in a 12-month program (February 2020-February 2021) using ED physicians to provide afterhours telehealth care in six RACFs.

**Methods:**

Using the NASSS framework, we synthesised descriptive analyses of statistical data from the MED service, RACFs and the ambulance service and a thematic analysis of interview data collected from GPs, RACF and MED service staff, and family members of residents.

**Results:**

Most calls to MED (179/209) were resolved with in-house treatment thereby reducing ambulance usage and hospital admissions. Interviews further revealed that MED enabled timely care for residents who were unwell but did not need hospital transfer. Technology, training, and rapid access to MED assisted RACF staff and complemented usual GP care. MED potentially reduced GP burnout. Refresher training was considered important especially in RACFs with high staff turnover, as was greater afterhours access to medications.

**Conclusions:**

The afterhours telehealth model provided in-house care and reduced ambulance transfers, and GPs and RACF staff generally felt supported. The service was easy to use and fostered good communications with GPs and RACF staff. Some GPs preferred to provide their own care, commenting on the need for a good understanding of patient and family needs and of the local context. Other stakeholders suggested this model could be extended to palliative care settings and to normal business hours when GPs were unavailable. The reduced ambulance and hospital use suggested benefits to wider health systems, however policies and funding that remunerate GPs, support community-based care and provide additional staffing in RACFs are needed to sustain afterhours telehealth in RACFs. Use of the NASSS (non-adoption, abandonment, scale-up, spread, and sustainability) Framework provided a valuable explanatory lens for our analyses.

**Supplementary Information:**

The online version contains supplementary material available at 10.1186/s12913-023-10257-5.

## Background/introduction

According to the recent Australian Royal Commission^1^ into Quality Aged Care, widespread reform is urgently required as the prevailing model of care in the current aged care system is largely reactive. Aged care services are not generally geared towards people’s enablement and do not maximise the maintenance and improvement of people’s health with services often unable to attend to complex patient needs. The Royal Commission noted that “many of the people and institutions in the aged care sector want to deliver the best possible care to older people, but are overwhelmed, underfunded or out of their depth” [1].

Access to care in Residential Aged Care Facilities (RACFs) is further compromised afterhours when there are fewer medical practitioners and RACF staff available and patients^2^ are often transferred to hospital emergency departments (EDs) for conditions that could be managed within the facility by primary care practitioners [2]. Electronic communication technologies provide a means of caring for patients in remote locations [3–6] especially as their speed and sophistication have greatly improved [7, 8]. Telehealth and video conferencing enable medical practitioners to diagnose and provide guidance for patient self-care and also for nursing staff who provide face to face patient care [9]. They also enable triaging of patients, escalation of care [5, 10], and electronic monitoring of patient symptoms in real time [11, 12]. These approaches have an important role in reducing the need for ambulance use, ED presentation and hospital admission as well as reducing medical practitioner workload afterhours [13–16].

Primary Health Networks (PHNs) are regional organisations funded by the Australian Government to improve coordination of primary health services in their region [17]. In 2020–2021, with funding from the Australian Government Department of Health and Aged Care, the Nepean Blue Mountains Primary Health Network (NBMPHN) implemented a 12-month telehealth initiative with six RACFs aimed at addressing the need for residents to have greater access to timely afterhours medical care and to reduce the transfer of RACF residents to EDs [18]. This telehealth initiative was implemented in the midst of rising COVID-19 infection rates in Australia alongside additional targeted telehealth initiatives to reduce face to face patient care and the risks of transmission of COVID-19 especially in the aged care sector and to reduce pressure on hospitals.

### Research aims and objectives

We aimed to evaluate the afterhours telehealth service from the perspectives of different stakeholder groups. We investigated its acceptability to key stakeholders, reported outcomes including hospital referrals, ambulance usage and in-house treatment, and provided recommendations for sustainability of the service.

## Methods

### Setting

The Nepean Blue Mountains (NBM) region west of Sydney, comprises four local Government areas including urban and semi-rural areas which cover almost 9,179 square kilometres [19]. The population in this region is aging at a faster rate compared to the rest of NSW with the increase in older persons as a proportion of the population, 5.13% compared to 3.3% across NSW between 2011 and 2026 [20]. There are 28 RACFs in NBM providing 2,622 full care residential beds [21] and 2148 independent living dwellings. All RACFs have 24-h access to Registered or Enrolled Nurses. More than half of all residents have a diagnosis of dementia. Most RACFs are not for profit (*n* = 24), and all offer respite care. Medical care is predominantly provided by GPs, however, health workforce shortages across the region affect access to GPs creating lengthy waiting times, especially afterhours [22].

In a trial program, for 12 months from 14 February 2020, the NBMPHN engaged the My Emergency Doctor (MED) telehealth service to provide afterhours consultations in six of these RACFs between the hours of 6.00 pm and 8.00am weekdays, before 8.00am and after 12.00 noon Saturdays, and all day/night Sunday and public holidays [23]. The MED clinicians are accredited Emergency Medicine Specialists with fellowship of the Australasian College of Emergency Medicine (FACEM). MED is a private organisation funded by fee for service. Staff at the RACF contacted MED on behalf of a resident needing acute care and liaised with the MED clinician. Consultations were conducted via a video app on an iPad [24].

Alongside the afterhours telehealth initiative, the Australian government introduced new temporary item numbers to the Medicare Benefits Schedule (MBS) for general practitioners (GPs) using telehealth including for aged care [25]. Medicare is the universal healthcare system in Australia and provides funding for GPs and other primary healthcare providers. The NSW government also introduced a secondary triage service for RACF staff calling triple zero (emergency) at any time, providing an assessment of the need for transfer and recommending either immediate ambulance support or a telephone consultation (also through MED) to manage care at the RACF [26].

### Study design

**Fig. 1:**
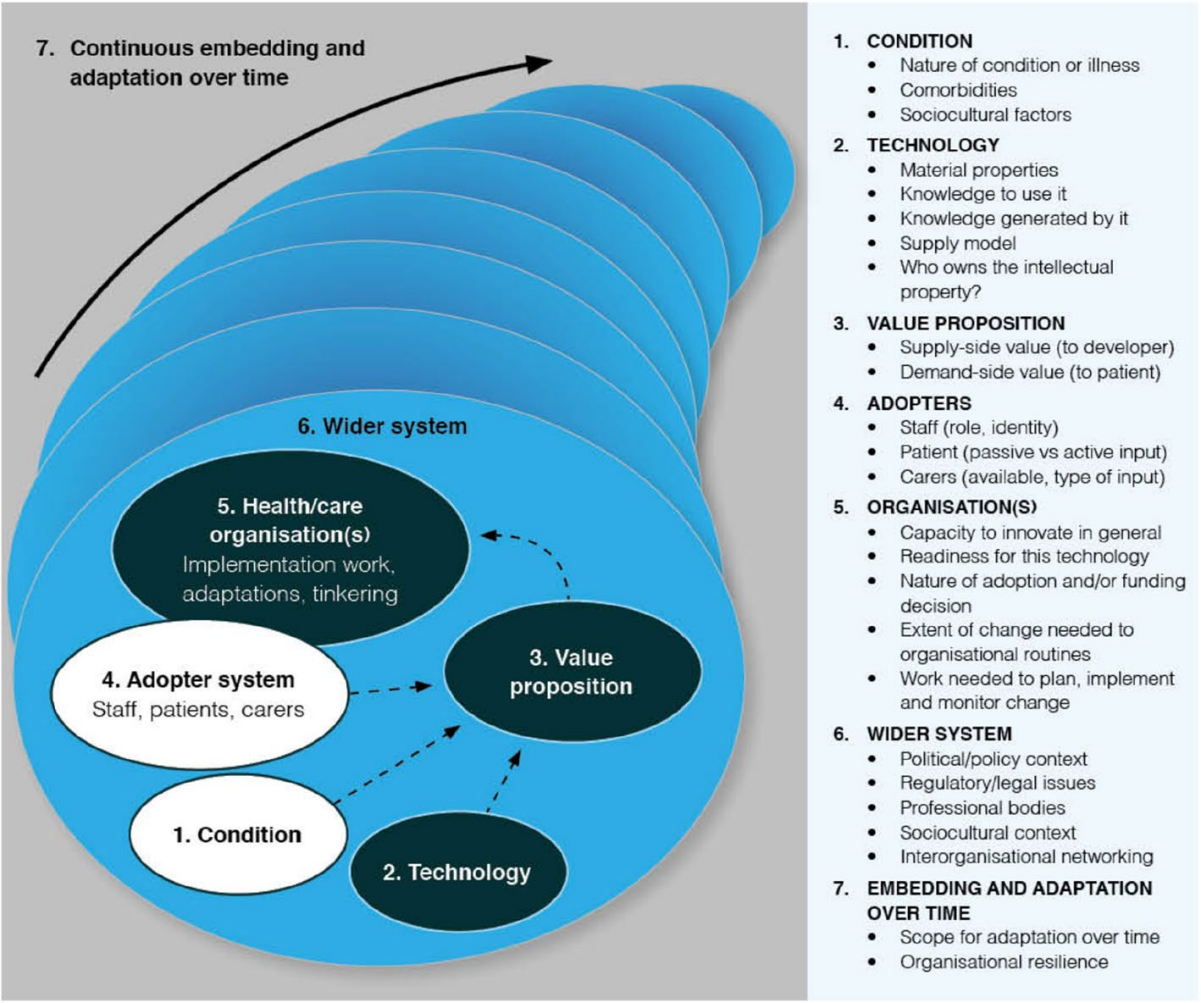
The NASSS framework for examining the influences on adoption, non-adoption, abandonment, spread, scale-up, and sustainability of healthcare technologies for patients and providers. Adapted from Greenhalgh et al. 2017 [27]

Our mixed methods study design included an analysis of quantitative data related to the use of the afterhours telehealth service and its outcomes, and also qualitative data collected through in-depth semi-structured interviews focused on participant experiences with the afterhours telehealth services. We then synthesised these analyses within an explanatory framework. The NASSS (non-adoption, abandonment, scale-up, spread, and sustainability) Framework provides a means of evaluating the adoption of health and care technologies through consideration of seven complex and interacting domains – the condition (problem) being addressed; technology being employed; the value proposition for stakeholders; the intended adopters; organisational capacity for change; the wider system; and, embedding and adaptation over time (Fig. 1) [27].

### Quantitative data collection

The NBMPHN established a comprehensive strategy for collecting data from the MED service provider and the RACFs for the afterhours periods between 14 February 2020 and 14 February 2021. The data included information on dates and numbers of calls, reasons for presentation, diagnoses, management, and recommended and actual usage of ambulance services for transport to an ED. The NBMPHN also provided data concerning individual RACF and GP engagement with the telehealth program that included the numbers of patients and GPs who consented to participate.

The NSW Ambulance Service provided historical data to the NBMPHN describing the total number of ambulance calls and ED transfers from the region’s 28 RACFs per year from 2017–2020 including separately for afterhours calls and ED transfers which were filtered according to the “afterhours” definition used for the telehealth program. The historical data did not cover the same timeframe as the MED service (February 2020 to February 2021). This data was reported separately to enable comparison between participating and other RACFs over that timeframe. Data collected during the MED service period did not differentiate the calls into NSW Ambulance and ED transfers where the call was transferred for secondary triage.

### Quantitative analysis

The NBMPHN provided the quantitative data to the Western Sydney University (WSU) researchers in a de-identified form for analysis. We conducted a descriptive and comparative analysis of the data.

### Qualitative data

Our qualitative data recruitment, collection and analysis processes were aligned to the COREQ framework [28] (Additional File 1).

### Research participants

We targeted a range of interview participants for the study. These included 8–10 staff from the six participating RACFs (nurses and managers), 8–10 general practitioners including both those who opted into the program and those who opted out, 5–6 MED staff (managers and clinicians), and 8–10 participating and non-participating RACF residents and their guardians. The NBMPHN identified and recruited a purposive sample of RACF managers and general practitioners by phone and email using an ethics approved letter of invitation and information/consent form. They ensured that all participating RACFs were represented and included managers and general practitioners who held different attitudes toward the program. The MED manager recruited clinicians and RACF managers recruited their staff, residents and guardians using the invitation and information/consent forms provided to them by the NBMPHN. Those willing to participate then contacted the WSU lead researcher (ST) to schedule an interview.

### Interviews

The semi-structured interview schedule was informed by a literature review as well as the program documents provided by NBMPHN and reviewed and revised in consultation with key NBMPHN program staff (Additional File 2). Interview questions explored participant experience of the afterhours telehealth program. Interviews were conducted either face to face or by telephone as preferred by participants, and audio-recorded and transcribed. Transcripts were checked against the recordings for accuracy and de-identified. Participants could review their transcripts if they wished. The interview schedule was piloted with each participant group and then minimally revised in consultation with the researchers as needed to ensure it remained focused but could also explore other emerging areas of interest.

### Analysis

We used an inductive thematic analysis to interpret the experiences and perspectives of participants with the afterhours telehealth program. This approach enabled us to describe patterns and meanings emerging from the interview data [29]. Research team members (ST, JR) each coded four of the first six interviews. An initial coding frame informed coding of the remaining interviews and regular meetings were held to check and refine the developing analysis and consider any differences in interpretation. Such a reflexive and collaborative approach to coding results in a richer more nuanced interpretation of the data [30]. At a final workshop, the researchers (ST, JR) reviewed all interviews and agreed that saturation of codes had been achieved. The final thematic structure was also agreed (Additional File 3). We then provided the thematic analysis to the NBMPHN program team for their input before finalising. After some clarifying discussion on one theme title, they generally agreed the themes and subthemes captured the data accurately. We used N-Vivo 11 ® software to organise interview data.

## Results

### Quantitative data analysis

#### MED telehealth service engagement

The six RACFs joined the telehealth service program between February 2020 and May 2020. The MED telehealth service received its first call on 14 February 2020 which was four days after the first RACF had completed its on-boarding process (engaging GPs and RACFs in the MED program). The time between RACFs being on-boarded and making their first call to MED varied from four to 133 days (x̄ = 41.7 days).

Most general practitioners providing patient care in the six RACFs (59/71) provided referrals for their full care residential patients to participate in the MED telehealth afterhours service. During the 12-month program period, 522 residents had referrals from their GP to access MED if required.

A total of 209 calls were placed by RACFs to MED in the 12-month period. There were almost as many calls for males (103) as females (106). The average age of the patients requiring MED was 83.6 years and the age range was 62–101 years. Two hundred and four of the 209 MED consultations were for patients of 18 of the 59 participating GPs. Five MED consultations did not have a GP recorded. Most of the calls to MED (175/204) were for patients of three of the 18 GPs. As patients were deidentified, we could not determine how many calls were provided for an individual patient. Table 1 provides engagement data.

**Table 1:** MED telehealth service engagement^a^

**RACF**	**Total residents in RACF (n)**^a^	**Total GPs in RACF (n)**^a^	**Referred residents (n)**^a^	**Referring GPs (n)**^a^	**RACF on boarding**	**Time to first MED call (days)**	**Total calls**
1	58	11	48	9	April 2020	61	2
2	144	21	131	18	April 2020	7	7
3	125	4	125	4	February 2020	4	169
4	68	6	47	3	February 2020	6	22
5	135	22	109	18	May 2020	133	7
6	68	7	62	7	March 2020	39	2
**Totals**	**598**	**71**	**522**	**59**		**x̄ = 41.7**	**209**

^a^Denotes maximums for GPs and residents as these numbers varied over time (for most RACFs up to 10%). For example, some GPs joined the telehealth program later than others

**Fig. 2:**
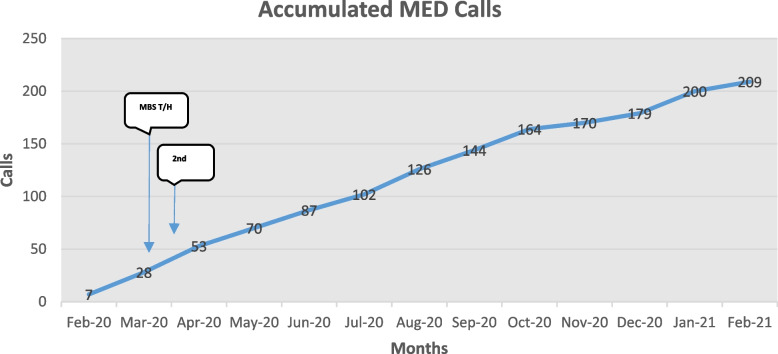
Accumulated monthly MED calls

Figure 2 shows the accumulated monthly calls to MED over the 12 months across the six RACFs. The timeline includes the commencement of MBS item numbers for GP telehealth (MBS T/H) and secondary triage (2^nd^) during COVID-19, since both of these programs are likely to have impacted on the data. Due to reported staffing limitations, only a minority (12%) of calls to secondary triage may have been transferred to MED.^3^

#### Presentations and diagnoses

The reasons for calls to MED by RACFs were recorded for each patient according to SNOMED categories. Table 2 provides the 10 most common reasons (162/209) for presentation according to higher order SNOMED categories [31].

**Table 2:** Reasons for presentation

Higher order SNOMED category	Subcategory according to SNOMED
Falls (95/209)	Elderly (85); mechanical fall (4); unwitnessed fall (2); minor fall (1); recurrent fall (1); falling injury (1); pushed over (1)
Pain (15/209)	Chest (5); abdominal (5); shoulder, neck, hip, headache, lower back (5)
Pharmacological assessment (11/209)	Medication review (10) and scripts (1) requested
Bleeding (8/209)	Haemoptysis (2); haematuria (4) blood in stool (1); rectal bleed (1)
Vomiting (8/209)	Vomiting (7); nausea (1)
Urinary (7/209)	Complication of catheter, blocked catheter, urethral discharge, urinary retention, reduced urine volume, dysuria, UTI,
Endocrine (6/209)	Hypoglycaemia (3); hyperglycaemia (1); diabetes mellitus (2)
Cardiovascular (5/209)	hypertension (3); hypotension (2)
Swelling (5/209)	Leg (2), toe, facial, neck
Fever (4/209)	(nonspecific)
Other (45/209)	

The 10 most common diagnoses provided by MED are provided in Table 3. These were mostly consistent with the reason for presentation; however, many diagnoses were more specific such as a presentation for feeling agitated diagnosed as schizophrenia, and fever often diagnosed according to the source of an infection.

**Table 3:** MED diagnoses

Higher order SNOMED category	Subcategory according to SNOMED
Falls (61/209)	Elderly (38); fall (14); mechanical fall (5); recurrent fall (4)
Injury (33/209)	Head (6); head minor (10); shoulder (1); soft tissue (2); contusion (2); tear skin (1); no apparent injury (11)
Pharmacological assessment (33/209)	Chart meds (28); script (3); chart and script (2)
Pain (14/209)	Chest (4); abdominal (1); shoulder (2) hip (2), migraine (1), lower back (1) post fall (1); knee (1); neck (1)
Urinary (9/209)	Complication of catheter (1); blocked catheter (2); injury urethra (1); UTI (5)
Vomiting (6/209)	Vomiting (3); nausea (1); coffee ground vomit (2)
Infectious disease (5/209)	Sepsis (4); clinical sepsis (1)
Endocrine (4/209)	Hyperglycaemia (1); diabetes mellitus (2); poor glycaemic control (1)
Disorder respiratory System (4/209)	Hypoxia (1); cough (1); lower respiratory tract infection (1); COVID risk assessment /flu (1)
Inflammatory Disorder (4/209)	Cellulitis (3); periapical abscess (1)
Other (36/209)	

#### Patient management

Most of the 209 calls to MED resulted in a recommendation for management within the RACF (*n* = 179). Thirty patient transfers to ED were recommended of which nine were recommended for Non-Emergency Patient Transport (NEPT). Table 4 provides the reasons for urgent ED transfers.

**Table 4:** Urgent hospitalisations

Reason for urgent ED Transfer	Residents (n)
Sepsis	4
Haemoptysis	2
Coffee ground vomiting	2
Infection, source unknown (with vomiting)	1
Periapical abscess	1
Gastric ulcer with haemorrhage	1
Gastrointestinal haemorrhage	1
Rectal haemorrhage	1
Cardiac syncope	1
Hypoxia	1
Lower respiratory tract infection	1
Post Operative (Cystoscopy) Bleed	1
Haematuria	1
Hip pain	1
Low back pain post fall	1

According to RACF data, there were 35 actual transfers to ED over the same time period with no explanation provided for this discrepancy. Further analysis revealed that 10 of the 179 patients recommended for in-situ management were actually transferred to the ED. However, of the 30 calls recommended for transfer to ED, five were managed in-situ. These discrepancies show that the RACFs did not always follow the recommendations of MED.

The RACF staff reported that in 87 of the 209 calls to MED, they would have normally called the ambulance service directly if the MED afterhours service was not available to them (Table 5).

**Table 5:** MED management plans

RACF	In-situ	Medication	Imaging	Pathology	GP Review	Recommended ED Transfers Emergency/NEPT	Actual ED Transfers	Cases where RACF would have normally called ambulance
1	2	0	0	0	0	0/0	0	1
2	5	3	0	0	1	1/1	2	4
3	147	23	2	0	12	15/7	26	58
4	17	7	0	0	4	2/3	6	18
5	6	0	0	0	0	1/0	1	6
6	2	0	0	0	0	0/0	0	0
**Totals**	**179**	**33**	**2**	**0**	**17**	**19/11**	**35**	**87**

Most patients (144/179) being managed within the RACF did not need specific treatment. A medication chart review or script was required for 33/179 patients and two requests were made for imaging. The MED service also recommended planning a GP visit for 17 of the 179 patients being managed within the RACF (in situ), however the urgency of GP review was not recorded (Table 5).

The MED service provided a consultation summary to the RACFs for all patient consultations. Table 5 provides the MED management plans in each of the RACFs. The table represents MED provided data except for the last two columns where the data was collected from the RACFs.

#### Use of ambulance service

**Fig. 3:**
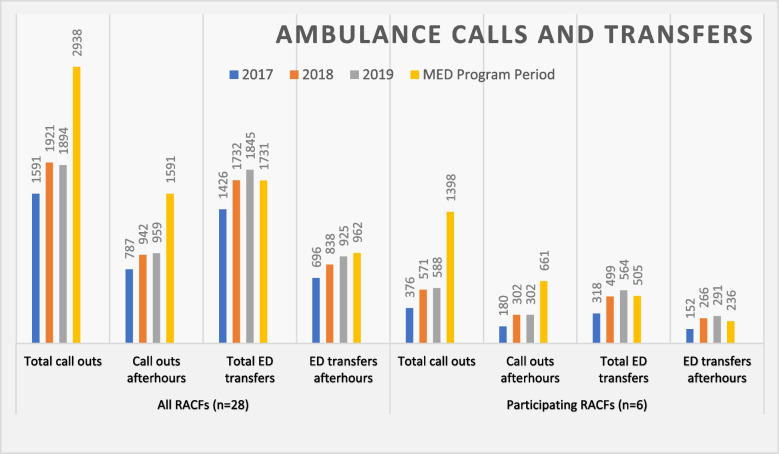
NSW Ambulance data 2017–2019 and during MED program period

The data provided by the NSW Ambulance service was filtered according to the “afterhours” definition used for the MED program. The number of afterhours calls into NSW Ambulance and ED transfers increased every year from 2017 to 2019 for the 28 Nepean Blue Mountains RACFs overall. The data collection period for 2020 was altered to fit with the MED program period (February 2020-February 2021). The six participating RACFs showed similar increases over these years but a reduction in afterhours ED transfers during the MED program period (Fig. 3). There is a large discrepancy between the ED transfers reported by participating RACFs in relation to patients referred to MED (Table 5), and the numbers of afterhours transfers recorded by the NSW Ambulance (Fig. 3), specifically 35 vs 236.

### Qualitative data analysis

We conducted 18 interviews between October and December 2020. The interviews were between 30–40 min each in length and included 7 RACF staff, 7 GPs, 3 MED staff and one guardian of an RACF resident (Table 6). Although we recruited for an additional month to 15 January 2021, we did not reach our target for any participant group and struggled particularly to recruit residents/guardians.

**Table 6:** Total interviews conducted

Participant Group	*“N”*	Target
RACF- Manager (RACF MG)	4	8–10
RACF Manager/Registered Nurse (RACF MG-RN)	2	
RACF- Registered Nurse (RACF RN)	1	
MED manager (1) or FACEM (2) (MED)	3	5–6
Participating GP (GP-P) = those opting in to MED	5	5–7
Non- Participating GP (GP-NP) = those not opting in to MED	2	3
Resident/guardian (RG)	1	8–10
**Total**	**18**	

Our thematic analysis of the interview data resulted in identification of four overarching themes. These were: Systems issues related to care in RACFs; Issues related to the MED Model of Care; Implementing the MED program; and Experience of the MED program. A number of sub-themes related to these are noted in Table 7 and described below with illustrative quotes. We provide the full analysis table in Additional File 3.

**Table 7:** Themes and subthemes

Systems issues related to care in RACFs	Issues related to the MED Model of Care	Implementing the MED program	Experience of the MED program
Challenges of delivering care in the RACF *-Resourcing including RACF funding and pressure on staffing numbers and time, and poor availability of medication*	Principles of management in RACFs *-Choosing the right locus of care* *-Team based care including residents and families*	Expectations of MED	Lack of GP engagement MED assists with specific needs
Some GPs often unwilling to provide afterhours care in RACFs	Scope of MED *-Perceptions of MED Role* *-Challenges with telehealth and role of video-health* *-Face to face contact* *-Complementary to usual care*	Promoting MED	MED program is reliable and provides valuable outcomes *-Communication from MED is efficient* *-Satisfaction of RACF staff*
	GP Model of Care Compared to MED Model of Care *-Local knowledge* *-Skill sets for RACF care* *-Continuity of care* *-Costs of service*	Process of implementing the program in RACFs *-Training* *-Consent* *-Privacy* *-Communications*	Improving the Afterhours MED program

#### Systems issues related to care in RACFs

Systems issues identified within this theme included challenges of delivering care in RACFs including inadequate funding and staffing, and access to medications, and also some GPs not willing to provide afterhours care in RACFs given the demands and poor remuneration for this work. This theme illustrated the experiences of providing care in RACFs before introduction of services such as MED.

##### Challenges of delivering care in the RACF

Interviewees often described the complex care needs of RACF patients and commented on poor funding for RACFs. These put pressure on staff and often resulted in delays in implementing instructions and providing medications. The general a lack of medications available in RACFs was also a source of concern.

the big challenge is there is not enough money in residential aged care … so the nurse-to-patient ratio is very low and that is a barrier…the nursing homes are 10 to one or 20 to one. Maybe two RNs on for 80 patients and the others are ENs, maybe seven or eight ENs. So 10 to one patient to nurse ratio as opposed to three to one in the public hospitals. (GP-P3)

These patients are sick, quite sick and really intensive. If I was seeing these patients in general practice each one would be my difficult patient for the day. Every patient at the nursing home is my difficult patient for the day. (GP-P3)

…I suggested that they should take his blood pressure more often and check his urine more often so we get a clearer picture, they obviously, haven't got the time to do that. (RG1)

the instructions and medications to give them, there’d be lot of delay by the time they implement it, so they will call you in the Sunday morning… but they won’t get the antibiotics until Tuesday. (GP-P5)

##### GPs often unwilling to provide afterhours care in RACFs

GPs reported feeling burdened with afterhours care and commented that RACF nurses frequently called about minor issues. Some declined to do this work especially when the remuneration was so poor. Without GP review, patients were said to be frequently transferred to the ED of the local hospital. However, GPs also noted that the Australian government funding of GP telehealth consultations as a result of the COVID pandemic provided them with a greater incentive to provide care in the RACF setting.

I don’t want to do afterhours, I don’t want to be in the middle of the night, as much as possible, I don’t want that (GP-P1)

They want to report every single thing; even minor things they report to you… It generally means that, for me, it’s taking a lot of my time (GP-P4)

Our nurses would normally ring the doctors and if we couldn’t get the doctors, and the clinical decision was that the resident was unwell and needed GP interactions, they would go into hospital, ambulance (RACF-MG2)

I'm much happier doing telehealth these days in the nursing homes… beforehand you're so bitter about all this telehealth that we did being effectively unpaid…but now if they [RACF] call me…I know I'm getting paid for it, I'll call them back and we'll go through it (GP-P3)

#### Issues related to the MED model of care

Comments included in this theme addressed Principles of Management in RACFs; Scope of MED; and GP Model of Care Compared to MED Model of Care.

##### Principles of management in RACFs

Interviewees noted the importance of choosing the correct location for care either at the RACF or in hospital. Deciding when to transfer to hospital was often described as challenging with the resident’s quality of life, the nature of the medical problem and risks for that patient of transfer of care (especially after hours) needing to be considered.

it’s quality of life around the residents because they’re not going into the hospital. They don’t have that disruption. Often when they go in an ambulance to hospital, they’re not taking sometimes dentures with them or glasses with them, just things like that, because everything is just quite rushed. So this way they stay in their home. Their quality of life while they’re just recovering from whatever the incident is or the deterioration is, it’s far healthier for them (RACF MG2)

Many interviewees noted the importance of working as a team that included residents and families, although, inclusion of relatives in the care team was observed by one interviewee as less well attended. Good decision making in multidisciplinary teams was described as relying on trust as well as good communication between team members, with a regularly reviewed advance care directive often a key component in that communication.

Everyone is involved in the care…it’s a chain of professionals that do the care for the residents. Obviously, at the front are the RNs and then it goes to the doctors and then next-of-kins (RACF MG4)

and I've suggested things that nobody seems to want to listen to me. Because I'm only a relative, sort of thing. And maybe I haven't got the right to do that, I don't know. (RG1)

we have a doctor’s book for the GPs that they look at every time they come. So they can see what we were wanting them to do for each resident, we’ve also got our handover sheet which gets discussed at each handover and as well as being documented in the progress notes and care plan (RACF MG6- RN3)

##### Scope of MED

Interviewees generally agreed that MED was more appropriate for acute afterhours care than for chronic conditions or when a procedural intervention was required. Used in this way MED reduced RACF staff reliance on GPs and the ambulance service.

Not for the chronic problems at all. It’s only meant for acute issues…purely meant to provide an opinion, advice in an emergency situation, really can’t do much for the normal case-to-case management in the long term at all. It has no role in that (GP-P5)

It’s being used after hours and where we would normally have rung an ambulance and/or a GP at this point. (RACF MG2)

Interviewees described challenges with telehealth and video-health consultations. They were concerned that MED did not have access to all patient records including medication lists and that an accurate assessment of the patient’s condition may not always be possible with telehealth. The video capability of MED was considered better than other forms of telehealth. However, it was also noted that MED communicated more with staff than patients.

That’s where I think there’s a lot of difficulty, when the patient is on 20 different medications and you’ve got a relatively junior nurse trying to read them all out to us. And the past medical history, it’s just very, very complex. That can be very time consuming (MED1)

I just worry because Telehealth is not 100% fool-proof, in the sense that some conditions really need to be assessed physically by a doctor to see what’s wrong with this patient – whether there’s a life-threatening condition or whether it’s just a simple thing. I’m just worried that one day the Telehealth doctors will miss something more serious, and the patient dies the next day (GP-P4)

they can actually speak to a doctor rather than talking over the phone. They can actually see the doctor and they can actually explain what’s going on and show the doctor the resident rather than just doing something by phone. (RACF MG6-RN3)

Many interviewees mentioned the need for face-to-face contact and some GPs commented on the importance of physical examination wherever possible, although this was sometimes impractical. Patients were said to prefer face-to-face contact with their GPs, but it was acknowledged that this was not always possible. Nurses and GPs considered the MED service complementary to usual care. It assisted their decision making and relieved some of the pressure they felt.

I personally like to do face-to-face medicine, not so much Telehealth, because you learn so much looking at the patient. And with Telehealth you can’t really get that idea from what they are in or other things they are describing. (GP-NP2)

they all love their GPs and they would prefer to see their GP, but it’s the difficulty of trying to get a GP out here when you need them. (RACF-MG2)

it [MED] has a big role to assist decision-making to the RNs [Registered Nurses] and the nursing staff. Even if it is emotionally taking the responsibility and the burden off the shoulders, it’s already a big role. (GP-NP1)

##### GP model of care compared to MED model of care

General practitioners highlighted the importance of their local knowledge including related to patient and family expectations. Nonetheless, the MED specialist emergency skills and training were also acknowledged as relevant to RACF work. Continuity across both the MED service and usual GP care was considered critical, and interviewees described ways of supporting continuity of care and mitigating risk.

it would probably be better delivered by GPs than emergency specialists … I just think GPs are better trained for nursing home work than emergency doctors are…It's community medicine, not hospital medicine that we're doing. (GP-P3)

we would definitely be complementing the face-to-face GP – it will always be necessary to have a local GP looking after a resident to have that continuity of care and ongoing management plan, so our service will never replace that and that’s definitely not our aim (MED3)

We’ve got a system where the senior clinical group, with MED, will audit the paperwork, a discharge summary and all their notes, to make sure that it includes everything relevant and necessary (MED1)

The costs of the MED service were an important consideration for many interviewees. They spoke about the additional costs of FACEMs and concerns about the sustainability of the service. One GP regarded the service duplicative if GP review was required following a telehealth consultation. Another questioned the legitimacy of providing MED with a 12-month referral in advance to enable Medicare funding for MED consults and expressed concerns that this could risk overservicing. This interviewee also noted that MED used primary care funding to the benefit of the (separately funded) hospital system.

it would be more cost-effective because we [GPs] don't bill as much as emergency specialists do. Even if you compromised and met them halfway it would still save a lot of money I would think…it's quite an expensive service. (GP-P3)

the after-hours Telehealth [MED] could occasionally be a duplicate service because they will ring Telehealth – I’m talking a lot of Telehealth consults is at night. And then the next day, when I come back, obviously I have to review the patient again the next day, I look at the report and I have to review the patient (GP-P4)

#### Implementing the MED program

Interviewees identified a range of issues related to the implementation of the MED program including their expectations of MED; the promotion of MED; and process of implementing the program in RACFs.

##### Expectations of MED

The GPs we interviewed expected MED would reduce their workload and the load on emergency departments. They also commented on the importance of MED clinical governance including clinical and medico-legal rigor.

really I expect them to call the whole after hours completely without me getting the calls in between (GP-P5)

I think my expectations are…it’s not just about the calls, it’s about the framework that we provide and medico-legal structure, follow up, access to notes. (MED2)

##### Promoting MED

Interviewees discussed the strategies used to promote MED to RACF residents and families, and highlighted shortfalls. The GPs expected RACF staff to promote MED to their patients, however one resident only became aware of MED through this evaluation. Lack of awareness of the program was also observed amongst GPs and sometimes also RACF staff who continued to call GPs afterhours. The MED provided ongoing orientation and training sessions to address the high RACF staff turnover.

We initially talked about it at resident meetings and we sent out a flyer, we put flyers up about it. And we also put it in our newsletter …to remind the residents …and the families, that that service was in place. (RACF-MG6 RN3)

I don’t know whether the nursing home or the staff are aware of the services, because…what I find is in the middle of the night, they would fax me about what is happening to this resident….I don’t know whether they are aware (GP-P1)

facilities with higher staff turnover –we do offer regular refresh training sessions for the staff, just so that any new members of staff who come through are aware of the service (MED3)

##### Process of implementing program in RACFs

Interviewees described how the program was implemented, commenting on the engagement and support, training, approach to consent, privacy safeguards, and communications processes.

The RACFs developed protocols for providing afterhours care through MED including clear definitions of “afterhours” and the process required by each GP.

most of the local protocols would still be for the nursing staff to phone the local GP in the first instance, and when they are not available, to then approach My Emergency Doctor. (MED3)

Challenges were encountered with engaging GPs. Some GPs preferred to provide their own care believing that afterhours care was their role and should not be commissioned from other providers. There was a belief that using the MED service was too time consuming for RACF staff.

initially he [another practice doctor] said, "Look, I'm not going to do it. I'm really pissed off. Bugger paying them. They can pay me." (GP-P3)

you need to speak to the nurse as the patient’s communicator most of the time to tell us exactly and that, I think, for some nurses… they feel like it’s a bit more time consuming. They need to spend time on the phone, looking through medications, talking to us, I think, they are feeling they can’t deliver care to other residents. I think they really feel a sense of pressure and rush (MED2)

Support from the PHN was critical in collecting patient consents and GP referrals as well as in establishing Information Technology systems in RACFs that supported implementation of the MED program. The MED service provided training for RACF staff who then trained others. These initiatives appeared to strengthen communications with MED.

they provided us with the iPad and then they did training with me specifically and I then trained my deputies and my RNs. (RACF MG2)

And we’ve done that again more recently because we’ve had new RNs starting and just to refresh all of us to make sure that we all remembered how to do it so (RACF MG6-RN3)

they would have had some training and would have had some expectations, I think, they use an ISBAR [Introduction, Situation, Background, Assessment, Recommendation] format, and so even when they speak, obviously they are pressured, I spend the first three minutes just listening and absorbing. I’ve never had a consult without vital signs (MED2)

Consent processes for the MED program and privacy safeguards respecting the dignity of residents and others were described by interviewees. These included de-identification of the evaluation data.

[RACF] has developed a telehealth policy, that’s only just come out a couple of months ago, around use of telehealth and confidentiality and stuff like that (RACG MG6-RN3)

You just have to make sure when people are recording a resident they are in dignified manner, the roommate is not being shown, it’s only focussing on what the issue in relation to that resident that they’re calling for (RACF MG3)

Communication was a high priority for MED and protocols were established early on. The RACFs and GPs received detailed reports on all consultations and clinical information was recorded in patient files and communicated to residents and families by RACF staff. The RACF staff also provided patient information to MED including advance care information. This bidirectional flow of information included GP health summaries provided to MED and MED care plans communicated back to GPs.

we got that in place, so everything is documented, the time that it was called, whatever ambulance has been called or after hour doctors have been called so that’s all been logged in. (RACF MG4)

we look at the advanced care plan and if it says palliative, not for hospitalisation, whatever, then we discuss that with the Emergency doctor, we talk to the family member as well, and the resident if the resident’s able to talk (RACF MG6-RN3)

we always send a clinical record or discharge summary to the aged care facilities, and a copy of that usually goes to the GP if we’ve got the GP’s number, and often if I’ve had the chance to speak to the GP looking after them, I will ask for their fax numbers. It’s not always readily available (MED2)

#### Experience of the MED program

This final theme addresses the experiences participants reported with the MED program. Interviewees highlighted variable GP engagement, MED assistance with specific needs, the reliability and value of the MED service; and suggested improvements in afterhours care in RACFs and the MED program.

##### Variable GP engagement

Whilst many GPs were relieved to no longer be on call for round the clock care, others declined to engage in the MED service or engaged conditionally. Generally, GPs considered MED as a complementary service to their care and one which could attract more GPs to nursing home work.

There were a number of GPs at each of the facilities who just point blank refused to sign any consent (MED-3)

We need to attract GPs to work in nursing homes and the after-hours service is actually an incentive because if you can say to GPs, "Well, you're not on call in the middle of the night, you're not on call 24/7 365 days a year," then it's much more attractive for GPs to work in nursing homes (GP-P3)

##### MED assists in meeting specific needs

Most interviewees regarded the care from MED as helpful, particularly as it enabled “in the home” care in the afterhours and acute care setting. The MED service was observed to address needs of the RACF resident population including those of varying cultures and non-verbal patients. Care providers felt supported in seeking to provide residents with good quality of life.

nursing home patients need 24-h care. If there is a case where I am away at night-time, they still can find someone if they have any problem and if there is any need of care, they can contact someone to review the patient, I’m happy. The patient is happy; the family are happy. (GP-P4)

I’ve been really surprised at how much we are able to make a difference without the patient leaving their home and without us leaving our home. That’s been, for me, a real surprise and makes it incredibly satisfying as a job. (MED1)

it’s [MED] a great app –very versatile for everyone.  Anyone can use it. I hundred percent love it and support it because it’s something that it can be used from toddlers right up to elderly and all culture and backgrounds (RACF MG4)

However, interviewees also reported MED being used inappropriately for repeat prescriptions of medications. They highlighted the importance of GPs rechecking prescriptions provided by MED.

We have been called for routine medications and that has created a bit of angst amongst us, but I see it is as if the patient or the resident does not have any other options. And for some reason, due to their aged care facilities, if it’s inherent busyness or their time constraints are unable to get a GP to fill out the medication charts, and I will just say look, I will just do it. (MED2)

They [MED]… might write down “I prescribe Endone” for a few days or weeks…. I usually don’t like to write S8 myself unless I feel that it’s necessary. I have to go and check because I didn’t prescribe (GP-P4).

##### MED program is reliable and provides valuable outcomes

General Practitioners reported that MED freed them to focus on priority patient needs and provided readily accessible, rapid care and advice. Follow up from MED reassured nurses and the video aspect of telehealth was particularly helpful.

I have to say that in the case of My Emergency Doctor, when they review a patient they direct your attention to what you need to review the patient because sometimes the patient may have a poly pharmacy and medication and they tell what you should do. So the general healthcare, the help is good because they direct your attention to what you need to do. (GP-P2)

… where the nurse is very worried, I’ve actually called back in three or four hours to check how the patient’s doing and I’ve found that just that one or two-minute call back after that, they found really reassuring, and it’s often the patient has picked up. (MED1)

it's great to have it there to know that we can ring somebody and they can actually visually see the resident after-hours if we need them, it's a great backup tool to have (RACF MG6-RN3)

Families appreciated care provided in the home which was faster than transferring to the ED. Many interviewees observed that MED reduced afterhours ambulance transfers to ED and hospitalisation likely resulting in health systems cost savings. There were many comments about the reduced burden and stress for GPs as well as residents and families.

feedback from the relatives or the guardians…from the nursing staff feedback, they always say, “Look, I’ve rung the guardian or the next of kin,” that this patient has been relieved by the after-hours Telehealth (GP-P4)

by the end of the day it saves time for the patient by having to wait for the emergency and gives them the service at the facility. (GP-P2)

It’s cost effective because it will save people going to hospital, use the resources or the ambulance because we know how expensive it is, and the hospital, stretching the facilities the emergency (GP-P2)

When they go to hospital, particularly if they remain in ED all day, they come back distraught. They come back upset. It’s an unsettling experience for them. And it’s not necessary when you’ve got something like My Emergency Doctor (RACF MG2)

The MED and RACF staff were observed to work well together and residents were included in a team-based approach. The MED clinicians enjoyed the challenge of providing care in the community and described supporting often isolated RACF nurses with the confidence to care for residents in the RACF.

One of the good things that have come out is it has improved my communication. It improves my emphasis of certain things. I need to think out of the box when I look at a patient, or how else can I provide care remotely. (MED2)

I found actually the nurses have been really happy – I personally felt a lot of positive feedback from the staff, and especially because it is out of hours and it must be quite isolating for the nurse. You know, they’re often one nurse, to a whole nursing home. (MED1)

So what it’s done is allowed us to just give the RNs the confidence that you can monitor them like you would normally do in hospital and then go from there (RACF MG2)

##### Improving afterhours care in RACFs and the MED program

Whilst interviewees generally expressed satisfaction with the MED service, they also suggested ways of further improving afterhours care in RACFs. Regular training in the use of MED was considered essential for RACFs with high staff turnover.

facilities with higher staff turnover – so re-engaging with the new staff, so we do offer regular refresh training sessions for the staff, just so that any new members of staff who come through are aware of the service (MED3)

Many interviewees offered practical suggestions to improve MED such as good internet connections and ensuring that MED clinicians had local knowledge. They also recommended increased use of My Health Record [national online summary and record of key health information for patients and health practitioners].^4^

I would love for [MED] doctors who know the locality and the area, it’s very important. I trust their medical knowledge, but one thing I am a little bit reluctant or hesitant is that they don’t have the knowledge of locality. (GP-NP1)

I think if there are some facilities which have the My Health Record and if there’s no opt out, the notes are on the My Health Record, that’s useful for the next clinician who sees the patient, whether it’s through the My Emergency Doctor or somewhere else to access the notes. (MED2)

The MED clinicians recommended improved access to advance care plans and directives to ensure appropriate care is provided. Some interviewees recommended extending of the MED service to palliative care and to cover routine office hours when the GP was unavailable.

I personally think palliative care via video consult with someone who is pretty sick or they are expected to pass away, I think there is value in us trying to save them to go to hospital. (MED2)

It would be so good if it was available throughout the day and we had our GPs on board to do that, then we could make a call through to them without having to present at ED, instead of waiting and chasing GPs to get things done and residents looked at, I think for me it’d certainly reduce day admissions. (RACF MG2)

Ongoing funding of MED as a complementary service to pre-existing community-based health services was recommended by some, although costs were recognised as a barrier to this. Others requested more funding for community-based services including for improved staffing in the RACFs, enhanced nurse practitioner models of care, and funded GP afterhours services.

My Emergency has been financially subsidised by the PHN. At the end of this trial or pilot trial any aged care facility who would like to continue on, will have to pay themselves, and the cost is not cheap – any future decision about continuity with financial sustainability has to be considered well. (GP-NP1).

…the actual thing we really need is more staff in the nursing homes. All that money could have been spent on some extra nurse practitioners (GP-P3)

It’s important I think to fund afterhours consults with GPs (MED2)

## Discussion

The NBMPHN commissioned the My Emergency Doctor (MED) service to address the need for timely afterhours care in its local RACFs. This 12-month telehealth program in six RACFs also aimed to reduce unnecessary use of ambulance services, ED presentations and hospital admissions, as well as the afterhours workload on general practitioners. This evaluation suggests the aims of this short-term program were largely achieved. Our findings and evaluations of similar programs such as the Hunter New England Partnerships in Aged Care Emergency using Interactive Telehealth (PACE-IT) study may inform other telehealth initiatives in RACFs [16].

In our discussion we have used the NASSS (non-adoption, abandonment, scale-up, spread, and sustainability) Framework to enhance our understanding of issues impacting on adoption of the MED approach to delivery of afterhours telehealth services in RACFs.

### NASSS Domain 1. The condition

The condition which was the focus of our evaluation was afterhours care in Australian RACFs. Many people living in RACFs have care needs that extend beyond assistance with day-to-day self-care. They require support that is not always able to be predicted and often requires skilled healthcare [1]. Our findings indicated that the care needs of RACF residents were often complex and included management of a range of co-morbidities. Residents could be taking as many as 20 different medications for these conditions. Interviewees reported that residents with dementia could not communicate well, and communication challenges were also noted when residents did not speak English as a first language or came from a different cultural background. Families often had differing expectations of the care being provided. This made care in this setting challenging especially for MED providers who didn’t know the patients and their families as well as the GP did.

### NASSS Domain 2. Technology

Relevant considerations in this domain include the material properties of the technology including its functionality, dependability, and speed, as well as the knowledge required to use it.

Telehealth services such as those introduced in the MED service have been shown to provide quicker access to primary and emergency care as well as intersectoral collaboration thereby avoiding patient deterioration and the necessity for hospitalisation [32, 33]. The recent Royal Commission into Aged Care recommended that “the Australian Government should require aged care providers delivering residential care…to have the necessary equipment and clinically and culturally capable staff to support telehealth services” [1]. In Australia, telehealth use was rapidly expanded during COVID, and the technology has become more accessible and familiar for those working in RACFs. Staff were provided with ongoing training on how to use the technology and on how to record patient care data. The video aspect of MED made using this technology attractive, although concerns were raised by our interviewees that use of telehealth may result in important signs being missed when the patient could not be physically examined. Interviewees noted that the technology was easy to set up but relied on good internet and Wi-Fi connections and may benefit from government investment in network bandwidth expansion and installation of higher generations of network technologies [34, 35].

The national electronic health record (My Health Record) [36] was suggested as a good way of sharing patient information including medications and advance care plans among care providers and improved use of this medium was recommended. This was particularly important for the emergency physicians from MED, who were frequently called for medication related issues. Similarly, it was noted that relaying complex medical information to MED by busy nurses was quite time consuming especially when nurse to resident ratios were insufficient. Beyond the RACF setting, it was noted that communications between health care providers across different settings in Australia are often suboptimal [37].

### NASSS Domain 3

The value proposition domain considered value to the patient, care provider, and healthcare system. Values considered in our evaluation included benefits of accessing the service, adhering to planned management, reduction in practitioner workload, complementarity with GP led care, cost effectiveness and opportunities for scaling the service.

Our findings indicated that those RACF residents who accessed MED received timely and appropriate afterhours care when their GP could not attend or be contacted. However not all residents, even in participating RACFs, had access to the MED service (when this was not approved by their GP), hence the limited reach of the program was a concern and may need to be addressed in future iterations of this program. During the 12-month program period there were 209 calls from the six RACFs to MED and our interviewees reported that calls placed to MED were usually answered immediately. In most cases (179/209), residents had their care needs attended to within the RACF. When similar in-situ care has been provided in other settings, resident outcomes and quality of life have improved [38, 39] and our interviewees also noted that such immediate, homebased care avoided the distress that residents and their families often experience with ambulance attendance and in-hospital care.

At times the MED planned management was not followed through with some patients transferred to ED who were recommended for in-situ care, while others were managed in the RACF despite a recommendation for transfer to ED. This may have been due to GP or family request contrary to MED recommended management, or a change in the resident’s condition.

Implementation of the MED telehealth service coincided with the outbreak of the COVID pandemic, and afterhours calls from RACFs to triple zero increased substantially at that time in both participating and all RACF groups. The increased number of calls may have been due to RACF staff being unsure how best to manage patients in that context [40]. The secondary triage service was introduced across all RACFs some two months after the MED program commenced, however information from NBMPHN program staff revealed that the triage service was not well utilised due to its staffing limitations and therefore when a triple zero call was made from an RACF, an ambulance may have been automatically despatched.^5^ This leaves us unclear on the contribution made by either the secondary triage or the MED program on the observed reduction in afterhours ED transfers in the participating RACFs.

Telehealth approaches have an important role in reducing medical practitioner workload and burden especially afterhours [13–15]. The GPs who referred their patients to the MED services expressed satisfaction with the reduced on-call work in the evenings or weekends especially knowing that their RACF patients would be well cared for. Many RACF staff interviewees also reported MED provided them with reassurance and support to enable more in-house care. They felt more confident with the team-based approach provided by MED who worked collaboratively with them.

The MED service used emergency specialists rather than a GP led model of telehealth but achieved similar outcomes to telehealth programs piloted in Australia in other contexts [12, 41]. Although many of those interviewed highlighted the importance of face-to-face care provided by a familiar GP it was acknowledged that use of the MED afterhours service could become complementary to usual GP care at times when GP care was difficult to access.

Telehealth in primary care contexts is considered cost effective especially through the reduction in ambulance use and hospital transfers [42–45]. The Australian Medical Association (AMA) has recommended urgent reforms in the aged care sector in order to save costs related to preventable hospitalisations [46]. Our findings both from the quantitative data analysis and the interview data demonstrated that the MED program reduced use of afterhours ambulance services and admissions to hospital. Some interviewees however, believed the costs of MED utilising FACEMs was much higher than if this care was provided by GPs. Others believed the MED services could be duplicative and even be open to exploitation. The costs for RACFs of staff training and supply and maintenance of equipment could also be a barrier in adopting these services [47].

Interviewees generally regarded the MED services as valuable for timely afterhours care in RACFs. The MED clinicians suggested the services could be scaled to other RACFs and also provided in office hours when GPs were working but may not be available for RACF calls. Some MED clinicians acknowledged that many aged care residents were at the end of their lives and suggested the MED service could be used to improve palliative care and thereby reduce unnecessary transfers to hospital. These comments aligned with findings from the Australian Royal Commission into Aged Care that too few people receive evidence-based end-of-life and palliative care, and instead often experience unnecessary pain or indignity in their final days, and that these services should be considered core business for aged care providers [1].

### NASSS Domain 4. The adopter system (staff, patients, carers)

Considering this domain we have reflected on how doctors and RACF staff engaged with the MED service and the importance of promotion, patient privacy and effective communication among care providers.

Engagement with the MED service was varied. Despite the recognition that an afterhours telehealth service was needed in RACFs, some GPs in the NBM region preferred to provide their own patient care in RACFs and declined to participate in the MED service. The role of non-GP specialists in providing community-based care was sometimes raised as a concern, in particular their lack of familiarity with local services and with patient and family needs. The importance of access to all relevant patient information including complex medication regimens was highlighted. However, some interviewees considered the MED model of care made work in RACFs more attractive to GPs as it provided afterhours cover. At a time when Australian GPs report reluctance to work in RACFs and RACFs commonly experience challenges in engaging GPs in care of residents, this is an important finding [48].

Similar differences in engagement occurred for the RACFs with some joining the service immediately, and others joining much later. Staff cited time constraints, a perception that the service was not needed and that their GPs provided adequate afterhours care. In other settings similar difficulties have been observed and the importance of strong promotion of the evidence for telehealth in this setting has been recommended [49]. Where the service was strongly promoted and supported by GPs many patients were referred to and managed by MED. Those GPs and RACF staff particularly noted the MED service as helpful and effective in managing a large number of patients afterhours. Interviewees emphasised that continued promotion of MED to GPs and RACFs as a complementary service and an opportunity to work together will be important, especially in those RACFs with high staff turnover.

Our interviewees spoke about the importance of privacy and dignity for patients and ensuring that data was de-identified. Effective communication between MED, RACFs and GPs was considered crucial. We noted that the implementation of MED prioritised these issues to the satisfaction of stakeholders which may have enhanced their engagement with MED.

### NASSS Domain 5. The organisation(s)

Relevant considerations in this domain included the disruption to existing routines, establishing the changes (including gaining buy-in) and evaluating the program.

Although the NBMPHN worked with RACFs and MED to establish processes for collecting consents, establishing protocols for privacy and collecting and reporting patient care data, for some RACFs the use of MED was a disruption from the usual practice of busy nurses calling an ambulance for patient support. Initially, RACF management had to remind nursing staff about use of the MED service as their first call because staff in the six RACFs frequently called triple zero instead of MED and a large number of patients were transferred to the ED as a result. Some RACFs commenced the MED program later than others and so prior to this (but within the overall program period) would have called an ambulance as their usual practice rather than MED. The RACFs did not need to report their ambulance use until they joined the MED program. This may partly explain why RACFs reported fewer afterhours ambulance transfers than the ambulance service (35 vs 236). Some GPs also reported that RACF staff were unaware of the service and still calling them afterhours. However, organisational change in the RACFs gathered momentum as the MED service became more established and familiar to staff. According to our interviewees and the RACF data, many of the calls to MED (87/209) would have normally been placed to triple zero instead. The benefits of the MED model of care may have provided an incentive for nursing staff to use this service, given their motivation to provide the best care for their residents [1].

Prior to implementing the MED service, a comprehensive data collection strategy was established by NBMPHN. This supported an evaluation that could inform the implementation of the program, identifying where changes needed to be made, and also informing its longer-term viability. We highlighted the need to improve the safety of medicine use, promote accountability, and improve decision-making in aged care and a focus on these areas has been recommended for stronger evaluation [1]. The evaluation of the MED service has identified how it worked for its stakeholders in terms of changing afterhours care in RACFs and how it could be improved, sustained, and scaled.

### NASSS Domain 6. The wider system

Considerations in this domain included cross-sectoral collaboration, government policy to support aged care needs, support from regulatory and professional bodies and health care funding models.

Collaboration between the non-GP specialist MED providers and primary care sector improved. The MED specialists were able to work with the RACF staff and attend to most patient needs in-situ thereby preventing many hospital transfers. MED also enabled access to ready medical advice for those who were unwell but did not require hospital transfer. Our MED interviewees reported how they were more connected with their RACF and GP colleagues despite use of MyHealthRecord not being adopted as routine in EDs and perceived by clinicians to be of more use for chronic and complex conditions [50]. Our interviewees described how prompt and accurate reporting and sharing of information enhanced patient care. Changes in clinical management could be implemented rapidly in the RACFs. Aligning with other research [33, 51], we found good communication and collaboration between MED, the RACFs and GPs supported continuity of patient care, and bridged the gap in afterhours services in RACFs.

At the time of implementing the MED service, the Australian aged care system was recognised as failing and was subsequently placed under the scrutiny of a Royal Commission [52]. The COVID pandemic was in its early days and disproportionately affecting aged care residents. The PHN, as part of its remit from the Australian government to work with GPs and RACFs, commissioned the MED service and its evaluation, however, there were no higher specific policy arrangements in place from government or other professional organisations to continue with this service approach. Although Australian government COVID responses included funding for GPs to use telehealth, this was not specific to afterhours work in RACFs. A need for increased funding and ongoing technical support from Australian governments has been identified to optimise the utilisation of telehealth in the aged care sector [53].

Strategies to reduce unnecessary ambulance and hospital use by RACFs were also introduced by the NSW government during COVID. The secondary triage service was a temporary policy to provide suitable care for patients at risk of contracting COVID. During this time, RACF staff were particularly susceptible to contracting COVID and when required to isolate placed greater demands on remaining RACF staff who did not feel supported in providing care to residents [54].

In a recent survey of RACF residents, 33% (129/391) believed their needs were rarely or never met [47]. Our interviewees described difficulties for residents accessing afterhours care prior to the MED service. There is strong support from the Australian public for significantly enhanced government funding in order to improve the quality of aged care in Australia [47] and the viability of services like MED will rely on this.

The RACGP has called for greater investment in GP led medical services for care provided in RACFs through blended funding models which incorporate fee-for-service, complexity loading and recognition for high-quality care [55]. Some GP interviewees considered the funding of the MED telehealth service contentious, stating that this funding should have been provided to GPs to adequately reimburse them for such afterhours care. Our evaluation also highlighted concerns about the Australian healthcare funding model more widely, whereby investment by the Australian government in primary health care resulted in benefit to the State government funded ambulance and hospital system. Others have advocated for review of this "split” healthcare system [56].

### Domain 7: Embedding and adapting over time

This domain addresses the sustainability and the ability of the program to adapt to the context, as well as the organisational motivation to continue using the technology and innovate through learning.

The technology being used by MED, particularly its video capacity, was well received and utilised. Once the RACF staff became familiar with the technology and the processes of using MED, it became easier for them to use. The availability and ease of use of the technology as well as ongoing training were key considerations of interviewees to continue using the service.

Sustainability depends strongly on funding and many of our interviewees were concerned about who would be responsible for ongoing funding of this program. They recommended that the MED service should continue as complementary to GP and other community-based care. Some interviewees suggested that the program could be expanded to include GP office hours and extended in scope to include palliative care. However, they highlighted the need to address the shortage of nursing staff in RACFs, especially afterhours. This observation is consistent with recommendations from the AMA to increase RACF nurse/patient ratios [46]. This is a critical factor without which ambulance call outs and hospital transfers will provide a quick, easy solution for staff who are overstretched.

### Insights from the NASSS analysis

Use of the NASSS Framework has provided a systematic review of our findings enabling a more nuanced understanding of the complexities of providing afterhours care in RACFs and the difficulties of implementing telehealth services at a time and within a healthcare sector confronting unique challenges. Influencing adoption positively, the “condition”, afterhours care in aged care facilities, is an acknowledged challenge in Australia and internationally [57, 58]. Our research adds to the evidence suggesting that telehealth is well suited to addressing this challenge when appropriate processes, orientation, training and support are provided [5, 41, 59]. The value proposition, in particular the cost savings in the acute care ambulance and hospital settings and reduction in GP afterhours workload, had a powerful influence on adoption of this innovation, as did the facility of the technology used. Although wider application of the MED program was recommended by some of those we interviewed, others questioned the costs and utility of the non-GP specialist model of care used. A key strength of the MED program was the attention to communication ensuring afterhours care was integrated with usual GP care. However, in spite of the strong organisational support for implementation of telehealth in the RACFs, wider systems issues impacted on uptake and influenced views about the sustainability of the MED model of care. These included inadequate staffing levels at RACFs, problems with medication management and concerns about funding models for community-based healthcare more widely. The sustainability of this and similar programs will require attention to these wider systems issues.

### Strengths and limitations

Collecting data from multiple sources strengthened the evaluation. The continued promotion of the evaluation among key stakeholders, and extending the recruitment timeframe, encouraged interview participation from most stakeholder groups including GPs who did not opt in to the afterhours telehealth service. The NASSS framework provided a valuable explanatory lens for our analyses. However, it was difficult to recruit residents and guardians as busy RACF staff could not assist with recruitment as expected, especially during the COVID-19 pandemic. We were unable to obtain detailed data that separated calls to NSW Ambulance and ED transfers that were managed through the secondary triage service implemented during COVID-19 or historical data that separated calls to full time residents versus independent living residents. The qualitative data collection concluded prior to quantitative data being provided which prevented further interrogation of this data through interviews. Although cost savings have been inferred from both quantitative and qualitative data, the cost effectiveness of the program was not studied.

## Conclusions

In an Australian context, the MED telehealth service helped address the afterhours care needs in participating RACFs and was considered by most stakeholders to be an effective means of providing care when face-to-face GP care was unavailable. With PHN and MED support, RACFs established policies regarding privacy, data collection, communication and sharing of information, although, in emergency situations accessing complex patient information was difficult sometimes and greater use of electronic health records was recommended. Adoption of the innovation by RACF staff improved as they became more familiar with MED technology and processes and recognised the benefits both for residents and staff. Residents received timely and appropriate afterhours care and there was a reduction in use of ambulance services and consequent ED attendance. Those GPs engaged in the program similarly recognised these outcomes and valued the reduced workload. However, not all GPs engaged, with some preferring to provide their own afterhours care and others expressing concern about a non-GP specialist led model of care. Whilst generally participants supported continuation of the service, and some advocated for its expansion, systems issues such as adequacy of RACF staffing and ongoing funding for community-based health care were noted as influencing the sustainability of the telehealth program. Policy changes at these levels will be needed as well as consideration of funding models that better support GPs to provide their own afterhours RACF care.

### Supplementary Information

**Additional file 1.**

**Additional file 2.**

**Additional file 3.**

## Data Availability

Raw data is not available for public access due to ethics requirements of privacy in place at the time of the initiation of this study. The authors declare that de-identified data supporting the findings of this study are available within the article and an additional file. Raw data are however available from the authors upon reasonable request and with permission of Nepean Blue Mountains Primary Health Network. Contact corresponding author Dr Steven Trankle s.trankle@westernsydney.edu.au.

## Abbreviations

AMA: Australian Medical Association
COVID: Coronavirus disease
ED: Emergency Department
FACEM: Fellowship of the Australasian College of Emergency Medicine
GP: General Practitioner
MBS: Medicare Benefits Schedule
MED: MyEmergencyDoctor
NASSS: Non-adoption, abandonment, scale-up, spread, and sustainability
NBMPHN: Nepean Blue Mountains Primary Health Network
NEPT: Non-emergency patient transport
PHN: Primary Health Network
RACF: Residential Aged Care Facility
SNOMED: Systematized Nomenclature of Medicine- Clinical Terms
Triple Zero (000): Emergency call number Australia
WSU: Western Sydney University

## Footnotes

1 Royal Commissions in Australia are the highest form of inquiry into matters of public importance https://www.royalcommission.gov.au/about-royal-commissions.

2 The terms “patient” and “resident” are used synonymously to reflect how they are referred to by the literature and participants cited in this research.

3 Email correspondence from NBMPHN.

4 https://www.digitalhealth.gov.au/initiatives-and-programs/my-health-record

5 Quantitative program data was provided to us after the conclusion of qualitative interviews and so this information could not be interrogated further.
