# Supplementary material for: Afterhours telehealth in Australian residential aged care facilities: a mixed methods evaluation
Source: BMC Health Serv Res. 2023 Nov 15;23:1263. doi: 10.1186/s12913-023-10257-5 (PMC10652444; doi:10.1186/s12913-023-10257-5)
Supplement: Supplementary file 2 — Additional file 2. [file 12913_2023_10257_MOESM2_ESM.docx]

**Additional File 2 Semi Structured Interview Schedules**

**All participants:**

*Thank you for agreeing to participate in this interview. I anticipate the interview will take approximately 30-40 minutes. The purpose of this interview is to understand how the new afterhours telehealth service (provided by My Emergency Doctor) in Residential Aged Care Facilities (RACFs) is working. We realise that a secondary triage service provided over the phone by the same organisation has also been made available since the start of the COVID pandemic for calls to NSW ambulance from RACFs. We are not evaluating that service. “There has also been GP access to bulk billing for telehealth services during this time”. You have been provided with a Participant Information Sheet and Consent Form. Have you read and understood these documents?* *Do you consent to this interview being recorded and your data being used in this project?*

*Do you have any questions before we begin?*

**Telehealth service provider (MyEmergencyDoctor-MED) – Manager and clinical staff**

*Prior experience and planning*

1. What experience do you have providing afterhours telehealth in RACFs? *(Manager and clinical staff)*

2. When you began the MED telehealth service pilot for afterhours care in NBM RACFs, what arrangements were already in place? *(Manager)*

Prompts: At your end?

In the NBM RACFs?

3. What needed to happen for you to deliver the service in the NBM? *(Manager)*

Prompt: How did NBMPHN assist you?

4. What links did you have in NBM with local health care providers? *(Managers and clinicians)*

Prompts: Staff at RACFs?

GPs, hospitals, other specialists, pharmacies?

5. How did these links need to be developed (explain)? *(Managers and clinicians)*

Can you tell me about protocols that were developed – what these were and who was involved in their development? (*Manager*)

Prompts:

- Staff training and availability/ rosters
- Ethical/legal concerns (incl. consent/ Privacy)
- Communications in RACFs, with GPs and with hospital and other services (incl. pharmacies)
- Technology availability, quality and maintenance
- Data collection (e.g. telehealth logs, ambulance calls, hospital transfers) and reporting.
- Patient management/ care planning

6. How were these protocols followed?

*Operations of the service (all questions for manager and clinicians)*

1. Could you please describe how (if at all) the contracted “afterhours” telehealth service provision in NBM RACFs has been impacted since MEDs implementation of the secondary triage service for ambulance calls coming from RACFs for COVID-19 and other conditions?

*We would like to understand how RACFs are using the MED telehealth service for afterhours care.*

1. Are some RACFs in NBM using MED telehealth more than others, or differently (explain)?

2. Is MED telehealth substituting for or complementing F2F GP consultations? OR GP telephone advice? OR services provided by the Local Health Districts specialist aged care team who also provide services via telehealth?

3. Have the new COVID Telehealth Medicare item numbers for GPs affected your provision of afterhours telehealth? If so, please describe the impact.

4. In what clinical settings is your MED telehealth service used? E.g. for routine, acute issues?

5. Have you found there are particular conditions, health issues that are suited to/ not suited to MED telehealth (explain)?

6. What sorts of residents/patients do you find MED telehealth useful/ not useful for?

Prompts: Do older patients or those with cognitive decline struggle with telehealth?

Has telehealth been delivered to Aboriginal and Torres Strait Islander people or

those from culturally and linguistically diverse backgrounds?

What particular considerations are needed for these populations (explain)?

7. How are decisions made about care?

Prompts: How are nursing staff or the residents/guardians, or the GP involved?

Are the RACF nurses sufficiently engaged and experienced to assist (explain)?

How confident are you in assessment of the resident by MED telehealth (given there may be limited pathology or radiology information available)?

How do you access/consider any advance care plan (ACP) the resident may have?

Prompt: How (if at all) do ACPs influence your decisions in recommending further care?

8. Have there been times when actual care was inconsistent with expected care (explain)? E.g.…

Prompts: With an ACP or directive?

With expectations of the RACF staff

With GP instructions?

With resident/guardian expectations?

*Communication (manager and clinicians)*

1. How do you communicate information about the advice you have provided back to the resident/guardian, RACF, and GP?

2. Are there any difficulties in communication (explain)? Or in collaboration?

*Outcomes (manager and clinicians)*

*A key purpose of the MED Telehealth service in NBM was to determine if more timely access to afterhours care could reduce patient deterioration, ambulance usage, or ED presentations and avoidable hospitalisations.*

1. Do you have any follow up with residents or hear back from staff about outcomes from the MED telehealth service?

What sort of outcomes can you report on (pos/neg examples)?

Prompts: For residents?

For RACFs?

Other?

Could anything else explain those outcomes beside the telehealth service?

2. Were your expectations of the MED telehealth service met (or not)? *(managers and clinicians)*

Prompts: What worked for you?

What other challenges did you face?

*For clinicians…*

e.g. difficulties resolving care issues within the RACF

e.g. RN capacity to assist (time, skills, language barriers, other)?

e.g. Is diagnosis and treatment more difficult than in a F2F consultation?

How do you address this?

e.g. How well can you guide patient self-care using telehealth?

*(Final questions for managers and clinicians)*

1. Are you aware of any unexpected benefits or unintended consequences of the program?

2. What recommendations could you make to improve the afterhours MED telehealth service in NBM?

*Thank you for your time today and the information you have provided. The transcript of this interview can be provided to you if you wish to check its accuracy. Would you like it made available to you? Is there anything else you would like to add before we conclude this interview?*

**Residential Aged Care Facility Nurses/Managers**

*Planning and implementation (nurses and managers unless specified)*

1. How were afterhours GP services being provided in your facility before MED telehealth?

How has this changed since implementing the MED telehealth service?

2. What did you have to do or change to assist in getting the MED telehealth service up and running in this RACF? *(Manager)*

3. What supports were provided by the NBMPHN to establish the MED afterhours telehealth service in the RACF? *(Manager)*

Prompts: What equipment (e.g. v/conferencing) was needed and how was this organised?

How important was this support in getting the service up and running?

4. What supports were provided by MED to establish the afterhours telehealth service in the RACF? *(Manager)*

Prompts: What equipment (e.g. v/conferencing), training was needed and how was this organised?

How important was the support from MED in getting the service up and running?

5. Can you tell me about protocols that were developed – what these were and who was involved in their development? *(Manager)*

Prompts:

- Staff training and availability/rostering
- Ethical/legal concerns (incl. consent/ privacy)
- Communications in RACFs and for GPs
- Technology availability, quality and maintenance
- Data collection (e.g. telehealth logs, ambulance calls, hospital transfers) and reporting.
- Patient management/ care planning

How were these protocols followed?

6. How is MED telehealth promoted to your residents/guardians? *(Manager and nurses)*

7. What challenges did the RACF face in implementing the MED telehealth service and how were these addressed*? (Manager and nurses)*

Prompts: E.g. afterhours staffing, language difficulties, staff expertise

8. Are there any ongoing challenges (explain)?

Prompt: Also ask about resident/ guardian understanding/ consent and *(for manager*) the protocols above.

*Operations of the service (Manager and nurses)*

1. *(use script to introduce and describe secondary triage)* Could you please describe how (if at all) the “afterhours” telehealth service provision in NBM by MED has been impacted since the implementation of the secondary triage service for ambulance calls for COVID-19 and now extending to all conditions?

Prompts: Are residents still receiving the timely and appropriate afterhours care they need? If not-why?

Has this impacted how afterhours telehealth services are provided by

MED? In what way?

2. *Use script to introduce and describe VACS.* Since the Virtual Aged Care Services (VACS) have extended their hours of support, has this impacted the provision/need of afterhours telehealth services by MED? How?

3. Under what circumstances would you use these other services instead of or as well as the MED afterhours service?

Prompt: E.g. when would you call triple zero instead?

*We would like to understand how RACFs are using the MED telehealth service for afterhours care*

1. Do some of your residents refuse MED telehealth? Why?

Prompts: Prefer F2F consultations?

Negative attitudes on telehealth?

2. When is MED Telehealth being used?

Prompts: Routine, acute issues?

3. Are there particular conditions, health issues that it is suited to/ not suited to (explain)?

4. Is it substituting for or complementary to F2F consultations? OR telephone advice?

5. What sorts of residents do you find it useful/ not useful for?

Prompts: Has MED telehealth been delivered to Aboriginal and Torres Strait Islander people

or those from culturally and linguistically diverse backgrounds?

What particular considerations are needed for these populations (explain)?

*Decisions and communication (Manager and nurses)*

1. Who usually initiates the MED telehealth consultation?

Prompt: How is that decision and its outcomes communicated to others (to whom)?

2. How are decisions made about care?

Prompt: How involved are nursing staff, the usual treating GP, and residents/guardians in deciding care?

3. How are care instructions or treatment plans from the MED telehealth provider communicated to the resident/guardian and GP and RACF?

4. How are any advance care plans (ACPs) the resident may have accessed?

Prompts: How is the ACP communicated to the MED telehealth provider?

How (if at all) do ACPs influence decisions in recommending further care?

*Outcomes (manager and nurses)*

1. To what extent has MED telehealth improved care for residents? How?

Prompts: Does it meet their needs (describe)?

Has their afterhours access to other health specialities improved (how)?

With whom (e.g. physiotherapists, pharmacists, hospital services)?

2. Were there any situations where care for the resident has worsened? (explain)?

Prompts: Continuity of care?

Communication between MED telehealth provider and the treating GP regarding treatment decisions?

Communication between RACF and GP?

3. Were there times when actual care was inconsistent with expected care?

Prompts: With recommendations from MED telehealth practitioner (explain)?

With an advance care plan or directive (explain)?

With GP instructions?

With resident/guardian expectations?

4. What have been the benefits of the MED telehealth service for the RACF?

Were there any negative consequences (explain)?

5. Are you aware of any unexpected benefits or unintended consequences of the program?

*A key purpose of the MED Telehealth service pilot was to determine if more timely access to afterhours care could reduce patient deterioration, ambulance usage, or ED presentations and avoidable hospitalisations.*

1. What outcomes can you report (pos/neg examples)?

Prompts: Have you noticed any changes in acute events (e.g. due to improvement in patient

health), patient self-care, QoL? Please describe these?

How big a part did the MED telehealth service play in these changes?

What feedback do you receive from families on outcomes of care?

Could anything else explain the outcomes and changes?

2. What recommendations would you make to improve the MED telehealth service?

*Thank you for your time today and the information you have provided. The transcript of this interview can be provided to you if you wish to check its accuracy. Would you like it made available to you? Is there anything else you would like to add before we conclude this interview?*

**Participating General Practitioners (opting in to MED service)**

*GP engagement*

1. Can you please describe your type of general practice (e.g. solo, group, corporate)?

1 a. Which RACFs do you provide services to?

2. How did you provide afterhours GP services in residential aged care facilities before MED and Telehealth?

And after MED and Telehealth?

3. What are the challenges of providing care in residential aged care facilities?

4. How did you hear about the MED telehealth pilot?

5. How were you involved in the MED telehealth pilot?

Prompts: Did you refer some or all of your patients (explain)? Proportion?

Did you refer those patients for all or only some health conditions (explain)?

6. Why did you participate in the MED telehealth pilot?

What were you hoping would be achieved?

7. What sort of changes did you need to make in your practice to participate in the MED telehealth pilot?

Prompts: Staffing (e.g. changes in hours, additional liaison)?

Changes in the care provided at the RACF? E.g. Changes in patient management (e.g. collaboration, communication)?

Data collection and recording/reporting?

Paperwork such as consents for referrals and reviewing and filing reports for each patient

8. How were you supported by the NBMPHN to participate in the MED telehealth pilot?

*Use of service*

1. Approximately what proportion of your RACF patients participate in MED telehealth?

2. How do you promote MED telehealth to your patients?

Are there patients to whom you don’t promote the service? Why?

3. What sorts of residents do you find it useful/ not useful for?

Prompts: Has MED telehealth been delivered to Aboriginal and Torres Strait Islander people

or those from culturally and linguistically diverse backgrounds?

What particular considerations are needed for these populations (explain)?

4. How are you using MED telehealth with your patients in residential aged care?

Prompts: Is it substituting or complementing F2F consultations? OR telephone advice?

Has this changed since Medicare rebates for telehealth consultations were

introduced?

Who decides to initiate the telehealth consultation with MED Telehealth?

How are your patients being offered MED consultation?

How does this work from your perspective?

When is it being used? Routine, acute issues, chronic?

Are there particular conditions, health issues that MED telehealth is suited to/ not suited to?

5. Does MED telehealth impact the relationship you have with your patients? How?

6. Can you describe your patients’ attitudes toward telehealth technologies?

7. Does MED telehealth impact the relationship you have with staff at RACFs? How?

8. Do you have any concerns about MED telehealth? Explain?

Prompt: Is privacy a concern for you? How?

9. *(Use script to introduce and describe)*. Are you aware of the secondary triage service (also provided by MED) for calls from RACFs into NSW ambulance?

10. Has this secondary triage telehealth service affected usual afterhours telehealth care? How?

11. *(Use script to introduce and describe)* Are you aware of the Virtual Aged Care Service (VACS) extending their hours of support due to COVID?

12. Has the extended VACS hours affected usual afterhours care? How?

13. Do the new telehealth item numbers for GPs affect how you provide afterhours care to your RACF patients? How?

14. Have the new telehealth item numbers for GPs impacted your view on the MED after hours telehealth service?

15. Under what circumstances would you use these other services instead of or in addition to the MED afterhours service?

*Communication*

1. How are care plans you have authored, or any ACP the resident may have, made available to the MED telehealth service (and RACF)?

2. How is the initiation of a MED telehealth consultation and its outcomes communicated to you?

Did this process meet your needs? If not, what was needed (e.g. a consult without your knowledge)?

*Outcomes*

*A key purpose of the MED telehealth pilot was to improve timely access to afterhours care for RACF residents and reduce ambulance use, ED presentations and hospital admissions.*

1. How has MED telehealth impacted on the care provided for your patients (examples if able to)?

Prompts: How well does it meet their needs?

Is the care timely?

Is the care provided appropriate in your opinion?

Changes in acute events (e.g. from improvement in acute condition)

What changes have occurred in ambulance and hospital use?

Have you noticed any changes in QoL for patients/guardians?

Could anything else explain those outcomes and changes?

2. Has the MED telehealth service enhanced patient access to other specialist services (how)?

With whom (e.g. allied HP, hospital specialists, pharmacists)?

3. Has MED telehealth impacted the way you are able to care for your patients (pos/neg)?

Prompts: Do you have any difficulties incorporating the care plan or recommendations from

the MED telehealth provider into your patient management (explain)?

How has continuity of patient care been affected (if at all)?

4. Were there times when actual care was inconsistent with expected care? E.g.…

Prompts: With your expectations (explain)?

With recommendations from the MED telehealth practitioner (explain)?

With an advance care plan or directive (explain)?

With patient/guardian expectations?

5. Were there any negative outcomes from MED telehealth for the patient (explain)?

*Impact on GP*

1. Was participation in MED telehealth cost effective/ ineffective for you (explain)?

2. How has your workload been affected through this pilot?

3. How were your expectations of the MED telehealth service met (or not)?

Prompts: What worked for you?

What challenges did you encounter?

4. Are you aware of any unexpected benefits or unintended consequences of the program?

5. Would you recommend the afterhours MED telehealth service to your patients?

6. What could be done to improve the afterhours MED telehealth service?

7. How if at all has the MED t/h service and/or the GP bulk billing t/h items changed your attitude / capacity / willingness to provide services to RACFs?

*Thank you for your time today and the information you have provided. The transcript of this interview can be provided to you if you wish to check its accuracy. Would you like it made available to you? Is there anything else you would like to add before we conclude this interview?*

**Non-Participating GPs (not opting in to the MED service)**

Can you please describe your type of general practice (e.g. solo, group, corporate)?

*GP provision of afterhours care*

1. How do you provide afterhours GP services in residential aged care facilities (e.g. F2F, telephone)?

2. Which facilities do you provide services to?

3. What challenges do you face in providing care in residential aged care facilities (explain)?

Prompts: How are you able to provide timely care to your patients?

Are there times when you are not available and the RACF has to initiate

management?

Can the RACF provide timely and appropriate care to your patients?

How well can you resolve patient issues within the RACF?

Including instructions to RACF RNs (and feedback to you)?

How well are you able to avoid escalation of care (e.g. ambulance use, ED/hospital presentation and admission)?

Provide care according to the expectations of patients (and guardians)?

Continuity of care?

4. Have you changed the way you provide afterhours care since the MED telehealth pilot began (explain how and why)?

5. Do the new telehealth item numbers for GPs affect how you provide afterhours care to your RACF patients? How?

Prompt: Have the new telehealth item numbers for GPs impacted your view on the MED after hours’ telehealth service? How?

5. *(Use script to introduce and describe).* Are you aware of the secondary triage telehealth service (also provided by MED) for calls from RACFs into NSW ambulance?

6. Are you involved in the MED secondary triage telehealth services being provided for patients?

Prompt: Has this impacted on your views about the afterhours MED Telehealth? How?

7. *(Use script to introduce and describe).* Are you aware of the Virtual Aged Care Service (VACS) extending their hours of support due to COVID?

Prompt: How are you involved in the VACS service being provided for patients?

How has this impacted on your views about afterhours telehealth?

*Non-participation in MED telehealth*

1. Where did you hear about the afterhours MED telehealth service?

2. Why did you decide not to participate?

3. Are there particular challenges for you in participating?

4. Have your patients (or guardians or RACF staff) asked you about including them in the MED telehealth service (explain)?

5. Where do you see MED telehealth fitting in to afterhours patient care in RACFs?

Prompts: Should it substitute or complement F2F consultations? OR telephone advice (or GP

Bulk Bill T/H)?

When should it (MED) be used? Routine, acute issues, other?

Are there particular conditions, health issues that MED telehealth is suited to/ not suited to?

Or patient types?

6. Can you describe any benefits (or negative outcomes) of the MED telehealth service that you are aware of?

7. If you were to participate, what support would you need?

8. What recommendations could you make to improve the afterhours MED telehealth service?

*Thank you for your time today and the information you have provided. The transcript of this interview can be provided to you if you wish to check its accuracy. Would you like it made available to you? Is there anything else you would like to add before we conclude this interview?*

**Aged Care Facility Residents and Guardians**

1. What was afterhours medical care like for you before the MED telehealth service (explain)?

Prompts: Was medical care provided when needed it?
 Was it the right care for you?

Was there a time when care did not meet your expectations (explain)?

2. How did you hear about the afterhours MED telehealth service? (RACF, GP, other)

3. Did you have any concerns about telehealth before you participated in the service?

What were they?

Prompts: Uncertainty about telehealth technology?

Privacy?

Changes in the relationship you have with your GP?

How were these concerns addressed (and by whom)?

*Use of MED telehealth*

*We would like to understand how you are using the afterhours telehealth service*

1.How often have you used the afterhours MED telehealth service?

Prompt: Once or twice or more often

Was this for flu/ COVID related concerns or for other concerns?

2. Did you get the care you needed through the MED telehealth afterhours service (example)?

Prompt: Did you get the care more quickly than usually?

3. Does your GP still provide treatment for you afterhours? How?

Prompts: In person?

by telephone?

through the RACF nurse?

*Communication*

1. How well does the RACF listen to you when they provide your care?

2. How well does the MED telehealth provider listen to you when they provide your care?

3. How well were the plans for your treatment communicated to you?

Or to your GP?

4. Do you have an advance care plan?

Did the care you received from the MED telehealth service match the information in your ACP (explain)?

*Outcomes*

1. What did you like about MED afterhours telehealth?

2. How well did the care you received meet your expectations (explain)?

Prompts: Did you receive care more quickly than previously?

Did the treatments provided work for you?

How could treatment have been better?

Did MED telehealth also look after other health concerns you had (explain)?

3. How do you think you have benefited from the MED telehealth service?

Prompts: Have you needed to use the ambulance less?

Visit the ED less?

Fewer hospital admissions?

How has the service helped you look after yourself (provide you with instructions)?

4. What don’t you like about using the MED afterhours telehealth service?

5. Will you continue to use the afterhours MED telehealth service if it was available?

6. Would you pay a fee to use this service?

7. What do you think would improve the afterhours MED telehealth service?

*Thank you for your time today and the information you have provided. The transcript of this interview can be provided to you if you wish to check its accuracy. Would you like it made available to you? As the guardian, would you also like the interview transcript made available to your resident* *should they regain capacity to check its accuracy? Is there anything else you would like to add before I close this interview?*

**Non-participating Aged Care Facility Residents and Guardians**

*Current afterhours care*

1. Does your GP provide treatment for you afterhours? How?

Prompts: In person?

by telephone?

through the RACF nurse?

*Non-participation in telehealth*

1. What is your understanding of the afterhours MED telehealth service?

2. Why did you decide not to participate?

3. Are there particular challenges for you in participating?

*Outcomes*

1. Can you comment on your satisfaction with the afterhours care you are currently receiving?
2. What could improve afterhours care for you in this RACF?

*Thank you for your time today and the information you have provided. The transcript of this interview can be provided to you if you wish to check its accuracy. Would you like it made available to you? As the guardian, would you also like the interview transcript made available to your resident* *should they regain capacity to check its accuracy? Is there* *anything else you would like to add before I close this interview?*
