# Supplementary material for: Afterhours telehealth in Australian residential aged care facilities: a mixed methods evaluation
Source: BMC Health Serv Res. 2023 Nov 15;23:1263. doi: 10.1186/s12913-023-10257-5 (PMC10652444; doi:10.1186/s12913-023-10257-5)
Supplement: Supplementary file 3 — Additional file 3. [file 12913_2023_10257_MOESM3_ESM.pdf]

Additional File 3

Thematic table: Qualitative evaluation NBMPHN Afterhours Telehealth Service in Residential Aged Care Facilities

KEY:
RACF-MG = RACF Manager
RACF-MG/RN = both roles
RN = RACF Registered Nurse
MED= My Emergency Doctor manager or FACEM
GP-P = Participating GP (opting in to MED Service)
GP-NP= Non participating GP (not opting in to MED Service)
RG= Resident/guardian

**Systems issues related to care in RACFs**	**Issues related to the MED Model of Care**	**Implementing the MED Program**	**Experience of the MED program**
Challenges of delivering care in the RACF<br>*-Challenges with availability of medication*	Principles of management in RACFs<br>**Choosing the right locus of care*<br>**Team based care including residents and families*	Expectations of MED	Variable GP engagement<br><br>MED fills specific needs
Some GPs often unwilling to provide afterhours care in RACFs	Scope of MED<br>**Perceptions of MED Role*<br>**Challenges with Telehealth and role of video-health*<br>**Face to face contact*<br>**Complementary to usual care*	Promoting MED	MED program is reliable and provides valuable outcomes<br>**Communication from MED is efficient*<br>**Satisfaction of RACF staff*
	GP Model of Care Compared to MED Model of Care<br>**Local Knowledge*<br>**Skill sets for RACF care*<br>**Continuity of care*<br>**Costs of service*	Process of implementing the program in RACFs<br>**Training*<br>**Consent*<br>**Privacy*<br>**Communications processes*	Use of other services<br><br>Improving Afterhours Care in RACFs and the MED Program

Systems issues related to care in RACFs	
Challenges of delivering care in the RACF<br>Complex patient needs	• the public hospitals are shifting all these quite ill patients into nursing homes without realising that the level of care is not the same… advanced cardiac failure or late stage pal care and cellulitis and dressings, extensive dressings that take a lot of time and a lot of nursing care to do… there's not that many nursing hours and doctor hours there to do that (GP-P3)<br>• These patients are sick, quite sick and really intensive. If I was seeing these patients in general practice each one would be my difficult patient for the day. Every patient at the nursing home is my difficult patient for the day. (GP-P3)
GPs are called for minor issues	• They want to report every single thing; even minor things they report to you. They ring me just for advice, even things like, "This patient has a bruise," or "This patient has a tear." They fall overnight or similar things. It generally means that, for me, it's taking a lot of my time (GP-P4)
Poor RACF funding and pressure on staffing numbers and time	• the big challenge is there is not enough money in residential aged care – just the overall funding, so the nurse to patient ratio is very low and that is a barrier…the nursing homes are 10 to one or 20 to one. Maybe two RNs on for 80 patients and the others are ENs, maybe seven or eight ENs. So 10 to one patient to nurse ratio as opposed to three to one in the public hospitals and doctors. (GP-P3)<br>• …when I suggested that they should take his blood pressure more often and check his urine more often so we get a clearer picture, they obviously, haven't got the time to do that. (RG1)
Nurses dilemma when GP and ambulance decisions conflict	• the poor RN is sitting in the middle with one way the GP is ordering to refer, to send to the hospital, and then if the RN rings the ambulance the ambulance will then question when it is not that 100% indicating to go. And then the hospital will then challenge the poor RNs and then the poor RN doesn't know what to do because they sit in the dilemma (GP-NP1)

Challenges with availability of medication<br>Delay implementing instructions and medications after hours	• the instructions and medications to give them, there'd be lot of delay by the time they implement it, so they will call you in the Sunday morning, will go and get antibiotics but they won't get the antibiotics until Tuesday again. (GP-P5)
Lack of available medications in RACF	• They [RACFs] don't keep all the stock of all the antibiotics …sometimes you may prescribe something which is not in their stock, then they have to wait until the chemist get them back again. By the time you post them and by the time they fax to the chemist, by the time they get it back, there could be delay of more or less 24 to 48 hours. (GP-P5)<br>• saving those [dying] patients a trip to the ED – some cases I've been able to palliate the patient in the nursing home, and all we did was prescribe morphine, midazolam, et cetera and they have that. Once it was a Saturday, I can't remember which nursing home it was, but they didn't have any stock and that just seemed such a shame that because of just the simple fact of them not having the medication locked up somewhere. (MED1)
Potential for increasing RACF capacity to provide extended care	• Day care is a short stay admission to the hospital. We are trying to avoid that by giving, for example, patients needing iron infusions; we write a prescription and get that iron and the RN can set up the drip up and do the infusion there. So they're also talking about allowing to use IV antibiotics but many times patients have to be transferred to hospital because they've got an acute chest infection. So they give IV antibiotics for two, three days and then send them back with oral antibiotics. So there is talk about being able to use intravenous antibiotics in the nursing home. It hasn't happened yet, but they are talking about it (GP-NP2)
Some GPs often unwilling to provide afterhours care in RACFs<br>GPs are burdened with AH care	• we were doing so much telephone work, enormous amounts every day, lots and lots of phone calls and being on call 24/7. (GP-P3)
GPs refuse to work after hours	• I don't want to do afterhours, I don't want to be in the middle of the night, as much as possible, I don't want that (GP-P1)

But some GPs only do RACF work and share the load<br><br>Other afterhours services can be utilised although with limited availability<br><br><br><br><br><br><br><br><br><br><br>Importance of advice from RACF staff<br><br><br><br>If GPs unavailable, patients referred to ED	• I don’t have private practice anymore, I’m one of the doctors who provides pretty well seven-days’ service [to RACFs]. If I’m away, then I get somebody to cover for me (GP-NP2)<br>• We would ring 136… an after-hours GP service and they actually come out to you, but we’ve found that their workload –that we would book an after-hours GP to come out and often more than not they would never turn up because they just got so busy even though you had an appointment booked they didn’t get out here (RACF MG6-RN3)<br>• I always work between 6.00 and 10.00 so I am always there until 10.00 so the only problem for me is between 10.00 and a few hours before 8 o’clock in the morning. But we used the after-hours doctors... Dial-a-Doctor or whatever, this is the service that we used to have – a deputising service. (GP-P2)<br>• I get some advice, some support from other staff members in cases of emergencies which reduces the risk of neglect for the patient, because you can’t – you are stretching the system by sending everyone that you can’t see to the hospital or get the after-hours doctor (GP-P2)<br>• Our nurses would normally ring the doctors and if we couldn’t get the doctors, and the clinical decision was that the resident was unwell and needed GP interactions, they would go into hospital, ambulance (RACF-MG2)<br>• we just sent to hospital, I just asked them, like the patient’s condition deteriorated or there was a change in their condition, then we just sent to hospital before the afterhours. (GP-P1)
Poor remuneration for GPs	• I'm still not very happy about it. I say, "Look, after hours, ring the after-hours service," because I'm not happy to be woken up at 2:00 am for $40 (GP-P3)<br>• I think some of the resistance that some of them [GPs] had mentioned in the past was they had to provide afterhours care and not be paid for it (MED3)

Poor MBS remuneration for work in RACF	• you can charge those item numbers only if I talk to the patient, but I'm not talking to the patient, I'm talking to the staff, so I can't charge for that… if the patient's guardian is in the room, I can charge the patient. If the guardian rings from home, I can't charge that. (GP-P5)<br>• I haven't got time to do care plans for 40 patients. I would love to be paid to sit in a nursing home and do care plans for my patients…I think you can't claim them on Medicare. So the care plan billing on Medicare, you're not allowed to claim it for nursing home patients (GP-P3)
Introduction of Medicare Telehealth items improve GP remuneration<br><br>Concerns about PHN funding role	• I'm much happier doing telehealth these days in the nursing homes… beforehand you're so bitter about all this telehealth that we did being effectively unpaid –it was unpaid before the telehealth was brought in that you would sort of avoid it or you would answer late (GP-P3)<br>• But now if they [RACF] call me, I usually call them back because I know actually, I'm getting paid for it, I'll call them back and we'll go through it (GP-P3)<br>• That's quite fair now in the sense that you might spend time advising them [RACF staff) and I do use it. (GP-P4)<br>• if the end point is to reduce hospital and ambulance admissions then I think the money shouldn't be coming from the PHN. The money should be coming from the hospital if that's their end point because they've got more money and I think the PHN money would be more effectively used by hiring more nurse practitioners or providing more community services (GP-P3)
**Issues related to the MED model of care**	
Principles of management in RACFs	
**Choosing the right locus of care**<br>Residents are better managed in the RACF	• it's quality of life around the residents because they're not going into the hospital. They don't have that disruption. Often when they go in an ambulance to hospital, they're not taking sometimes dentures with them or glasses with them, just things like that, because everything is just quite rushed. So this way they stay in

	their home. Their quality of life while they're just recovering from whatever the incident is or the deterioration is, it's far healthier for them (RACF MG2)<br>• the whole experience of being transferred from an environment that they feel safe and comfortable in to going into Emergency Department where often they would need to wait for a couple of hours before actually being seen to, so I believe that there was a gap in that particular area (MED3)
Hospital can be best place in some circumstances including when residents or families prefer	• one resident where the emergency doctor said that we need to send him off because he really needs to be transfused, or whatever. Or the catheter needed to be changed, or needed to be given IV antibiotics in the hospital, instead of them being septic in here (RACF MG3)<br>• 000 is called when for example someone's fallen and there is a cut in the head, someone has fallen and you can see that one leg is shorter than the other (RACF MG4)<br>• Somebody collapsed on the floor, they just call for an ambulance. I don't think they'll triage with the afterhours doctor, they don't (GP-P5)<br>• it's very few. But these are like, let's say, traditional people who think that the hospital is the best place for someone if something happens to them. (RACF MG3)
Challenges of deciding when to transfer care to hospital	• like all doctors, the art is to predict whether it's going to get worse, and the decision-making will then be, is that something that is manageable and can wait until the morning or is it something that is going to escalate and be too much for the patient or for the best practice management criteria. Then that's the decision-making (GP-NP1)<br>• I just break it down as to, do they need to go to hospital today? If the answer is yes, would they benefit from going to hospital today? And then once I've figured that out then it doesn't really matter. We don't really need to know if they had a mini-stroke on the Saturday or the Friday…especially for the patients that have

	already had strokes and already on aspirin because, they're already on maximal medical therapies. Sending them in on a Saturday night isn't necessarily going to add to their quality of life or add to anything really. (MED1)
	• there's a spectrum as well. You could have a very minor injured older person and then the nuances obviously, we have to balance the risks of transferring someone with maybe cognitive impairment at one to two am versus could we wait a little bit until six or eight o'clock in the morning (MED2)
**Team based care including residents and families**<br>Importance of resident and family involvement in decisions	• if the resident is able and capable, then obviously they make that decision in conjunction with the doctor and the care team, or if they cannot decide for themselves obviously. We get in touch with the doctor, Public Guardian or the nominated Power of Attorney or person responsible (RACF MG1)<br>• when required, case conferencing by phone or sometimes we do have face-to-face meetings with one of the children or sometimes with two if required. And we then have a good discussion about what are their expectations. We explain to them what the condition is, what the prognosis is, what are the medications. (GP-NP2)<br>• and I've suggested things that nobody seems to want to listen to me. Because I'm only a relative, sort of thing. And maybe I haven't got the right to do that, I don't know. (RG1)
Care is collaborative and multidisciplinary	• Everyone is involved in the care…it's a chain of professionals that do the care for the residents. Obviously, at the front are the RNs and then it goes to the doctors and then next-of-kins (RACF MG4)<br>• Obviously, we're utilising dieticians and we're doing pharmacy and all that stuff through Telehealth as well (RACF MG4)<br>• I work hand in hand with them, and I know the people quite well. The nurses that come to the facility, I work with her – there's one nurse that I know so well that we work together. (GP-P4)
Trust in other members of the team underpins good decision making	• you have to inherently trust, if nurses give us the wrong vital signs there could be trouble. I guess trust goes both ways. They are

Continuity of care in a team based care model<br><br>Current Advance Care plans for all RACF residents	receiving instructions or reassurance from us and we have to receive, so radiology and pathology, most of clinical medicine, I guess we rely on a good history or if possible a good video look, looking at the patient (MED2)<br>• we have a doctor's book for the GPs that they look at every time they come. So they can see what we were wanting them to do for each resident, but we've also got our handover sheet which gets discussed at each handover and as well as being documented in the progress notes and care plan (RACF MG6-RN6)<br>• I just forward email communications, letters on what stage we are at with our process. And disseminating this information. Is being added in her (RN) end shift report which is provided to all the registered nurse team including the Care Manager and myself, of the General Manager. And then the registered nurse is also updating the family members or whoever is the guardian or if ok for the resident (RACF MG1)<br>• Simple things like expecting every patient to have an advanced care directive and have their advanced care directive reviewed every three months. (GP-P3)<br>• I would say 95% of my patients do have one. They call it a Care Directive. (GP- P4)<br>• we're very on top of that, advance care directive are being sent together with records to the hospital…it's also recorded on our computer system, it's an electronic system, documentation system (RACF MG1)
Scope of MED	
**Perceptions of MED Role**<br>GPs perceive MED Telehealth is a service for them not RACF patients	• it's not really a service for the patients. It's a service for the doctors. So really, it's not being provided for patients because we were providing the service previously. It's mainly a service — I don't think anyone is selling it to the patients because it's not the patients making decisions. It's the nurses and the doctors that make decisions about whether we're going to use it. (GP-P3)

Afterhours only	• the whole idea of us being involved is to be the primary care for emergency after hours care (MED2)<br>• It's being used after hours and where we would normally have rung an ambulance and/or a GP at this point. (RACF MG2)
Acute care rather than ongoing management	• Not for the chronic problems at all. It's only meant for acute issues…purely meant to provide an opinion, advice in an emergency situation, really can't do much for the normal case-to-case management in the long term at all. It has no role in that (GP-P5)<br>• they're useful for emergency calls after hours basically. Palliative care, probably not so much because the palliative care are all expected to go downhill so we plan it out. In general it's not suited to most chronic health condition problems. It's only really suited to acute health conditions (GP-P3)
MED suited to many but not all emergency conditions	• Suited to all of the emergencies that we have, like patients who have falls or need to make a decision about to send them to hospital or not to do a scan, the patient has got confusion or delirium, and the patients have got chest pain, the patient has abdominal emergency, acute abdominal. So all of these emergencies, are suitable for me for the Telehealth doctors to contact and make a decision based on the information available from the staff, and from the patient as well (GP-P2)<br>• I'm an ex hospital DON [Director of Nursing], so there's no way that we can manage an aspiration here. We don't have high flow oxygen (RACF MG2)
**Challenges with Telehealth and role of video-health**<br>MED may be unable to access all records	• there's a big issue with communication in that emergency doctors don't have very good access to the records, the patients' records. So they can't really understand what's really going on with the patients. (GP-P3)
Telehealth may not provide an accurate assessment	• I just worry because Telehealth is not 100% fool-proof, in the sense that some conditions really need to be assessed physically by a doctor to see what's wrong with this patient – whether there's a life-threatening condition or whether it's just a simple thing. I'm

	just worried that one day the Telehealth doctors will miss something more serious and the patient dies the next day (GP-P4)
Difficulty charting medications but new software is helpful	• the only challenge that we got, was that – the charting of the medications if need be (RACF-MG3).<br>• That's where I think there's a lot of difficulty, when the patient is on 20 different medications and you've got a relatively junior nurse trying to read them all out to us. And the past medical history, it's just very, very complex. That can be very time consuming (MED1)<br>• The challenges are the medications, which is one thing that you need to supply them with this prescription, but now with the software, it's easier. (GP-P2)
MED provides a video benefit to telehealth	• they can actually speak to a doctor rather than talking over the phone. They can actually see the doctor and they can actually explain what's going on and show the doctor the resident rather than just doing something by phone. (RACF MG6-RN3)
Telehealth communication is mostly with staff than with patients	• you're talking about patients – 50% of my patients have dementia, so they can't communicate for that reason…or Parkinson's disease. I think there's a whole lot of reasons that people in nursing homes can't communicate. Advanced cerebral palsy, all these sort of issues. (GP-P3)<br>• Some of the patients, they're cognitively fine, whereas in this case a lot of them have the nurse with them, so then most of the instructions go to the nurse really, in the nursing home (MED1)
**Face to face contact**<br>Some GPs prefer to physically assess patient	• even though you might be able to see the patient it's not the same as being there with them. (GP-NP1)<br>• let's say I have a patient with acute abdomen and then they say, "Okay, give them some Panadol." After of course having the consultation with the staff, and things like that. I don't think acute abdomen should be treated like that, and this is why then I went over there and examined the patient. Because I didn't think that this is the right thing to do. (GP-P2)<br>• I personally like to do face-to-face medicine, not so much Telehealth, because you learn so much looking at the patient. And

Some GPs say that F2F is not needed in many cases<br><br>Impractical to do all consults F2F<br><br>Residents and families prefer face to face care	with Telehealth you can't really get that idea from what they are in or other things they are describing. (GP-NP2)<br>• I know that in many of the cases, by having the telephone call, I don't think that the patients need to be seen personally face-to-face. (GP-P2)<br>• they all love their GPs and they would prefer to see their GP, but it's the difficulty of trying to get a GP out here when you need them. Obviously, they're all in general practice as well. So it can be quite difficult to do that. Obviously, they'd love to see their doctors more, but it's not within reason (RACF-MG2)<br>• most of our patients prefer face-to-face, they prefer to talk to you and discuss with you what they have. Especially with our senior community. (GP-P2)<br>• challenges are to be able to see the patient or to make decisions about sending them to the hospital or not, because sometimes families insist on getting the doctor to see the patient. (GP-P2)
**Complementary to usual care**<br>Assists nurse decision making<br><br>Complements GP care	• we're using the After-Hours Emergency – every time after 5 o'clock when the doctor is not available and when the registered nurses are feeling, they need more support and assistance for the medical and clinical intervention of the resident. (RACF MG1)<br>• it [MED] has a big role to assist decision-making to the RNs and the nursing staff. Even if it is emotionally taking the responsibility and the burden off the shoulders, it's already a big role. (GP-NP1)<br>• we're complementing them [GPs], so … the GP might have sent off a urine test on the Thursday, but the result didn't come until the Saturday morning, and then we can be reviewing the patient and prescribed the antibiotic (MED1)
GP Model of Care compared to MED Model of Care	
**Local Knowledge**<br>GPs know patients and local services	• if you know the person it's a lot easier to treat otherwise, it takes a long time to treat especially on the phone, it's impossible to advise on the phone unless you know that patient, unless you know the family…what the family expectations are. Teleconsultation without knowing the patient is not easy in the aged care setup (GP-P5)

	• they're [NBMPHN] getting these doctors who don't know the patients to review the patients. It's not like when I come in, I really assess the patient, I know them, I've got a relationship (GP-P3)<br>• I'm not happy, that My Emergency, he might be in Melbourne, so when the patient is in Springwood, the decision would be go to Katoomba or go to Nepean Hospital, and he would not know that Katoomba would be lovely for our older patients. (GP-NP1)
**Skill sets for RACF care**<br>GPs more suited to RACF care than emergency specialists	• it would probably be better delivered by GPs than emergency specialists because despite emergency specialists being – I just think GPs are better trained for nursing home work than emergency doctors are…It's community medicine, not hospital medicine that we're doing. (GP-P3)
MED has specialist emergency skills	• the emergency doctor, you feel comfortable that you have experienced people who will take care of the concern of the nursing staff and patients. Because they are specialists dealing with this situation. They are trained by emergency, which is what the patient is going to see because I understand that this emergency specialist will manage it (GP-P2)<br>• I don't think all – a lot of GPs would be – would have that skill set. We've always – a lot of our staff with at least 10 years of hospital training in emergency medicine, but some of the other consultants they're in their mid-50s or older, so huge amounts of hospital experience. (MED 1)
**Continuity of care**<br>MED provides continuity of care	• the resident remains in the nursing home, there's still continuity of care. He or she doesn't feel that they're strangers, different strangers again looking after me (RACF MG3)
Importance of GP in continuity of care	• we would definitely be complementing the face-to-face GP – it will always be necessary to have a local GP looking after a resident to have that continuity of care and ongoing management plan, so our service will never replace that and that's definitely not our aim (MED3)
Mitigation of risks to continuity of care	• We've got a system where the senior clinical group, with MED, will audit the paperwork, a discharge summary and all their notes, to

**Costs of service**<br>Additional costs of FACEMs<br><br>Sustainability<br><br>Telehealth may be duplicative<br><br>Legitimacy of use of Medicare funding for MED questioned	make sure that it includes everything relevant and necessary- all of us understand that that is our legal – it sounds bad but it's the legal record for continuity of care for the patient, it's our legal defence. (MED1)<br>• it would be more cost-effective because we [GPs] don't bill as much as emergency specialists do. Even if you compromised and met them halfway it would still save a lot of money I would think… it's quite an expensive service. (GP-P3)<br>• after March next year, if the after-hours doctors still can be paid by the Telehealth, or are they going to be paid by the PHN or what? (GP-P4)<br>• My Emergency has been financially subsidised by the PHN. At the end of this trial any aged care facility who would like to continue on, have to pay themselves, and the cost is not cheap because of course the fees of the Medicare does not cover the whole bit… any future decision about continuity was financial sustainability (GP-P3)<br>• the after-hours Telehealth could occasionally be a duplicate service because they will ring Telehealth – I'm talking a lot of Telehealth consults is at night. And then the next day, when I come back, obviously I have to review the patient again the next day, I look at the report and I have to review the patient (GP-P4)<br>• Medicare specialists need to have a referral letter. So these guys are gaming the system a little bit. … So to bill, they ask us to write a referral at the start of every 12-month period and it's an open referral to see them for emergency issues and we provide a little summary. (GP-P3)
**Implementing the MED program**	
Expectations of MED<br>GPs expected their workload to be reduced through fewer calls<br>Reduce load on ED	• really I expect them to call the whole after hours completely without me getting the calls in between (GP-P5)<br>• I think with Telehealth, actually, my expectation is to reduce the over-crowdedness of the emergency centre. (GP-P4)

All TH consultations should be clinically and medico legally rigorous	• I think my expectations are…it's not just about the calls, it's about the framework that we provide and medico-legal structure, follow up, access to notes. I just would prefer, it would be nice if all telehealth consults can be a face to face, like using an app or using an iPad to look at (MED2)
Promoting MED<br>Strategies for promoting to residents and families	• We initially talked about it at resident meetings and we sent out a flyer, we put flyers up about it. And we also put it in our newsletter …to remind the residents …and the families, that that service was in place. (RACF-MG6 RN3)<br>• We let them know during our family case conferences, because you also have to get consent if they have been – before we send anyone to hospital. (RACF MG3)<br>• We are informing the family as well. Let's say, during case conference or, let's say, we were observing that this resident, that they have some medical concern. We are informing the family as well, via phone calls, that we are using this one as well, in case that the doctors are not available after-hours (MG5 RN1)
A resident only found out about MED through the evaluation	• Well, I didn't know about the after-hours telehealth until they handed – well, they actually handed these sheets from Western Sydney University to my son, who handed them to me. I read through them and I thought, well, that would probably be the first time I'd really know about the after-hours telehealth service (RG1)
GPs rely on RACFs to promote	• I don't personally promote it, because most of our patients have dementia, have problems with communication. So promotion is really through the nursing staff, the Care Manager. (GP-P4)
Poor GP understanding	• I didn't really know that it is a special service, this Emergency My Telehealth, I thought that the nurse was just able to speak to the ED doctor and was given the advice…I don't know whether it's just me or other GPs providing care have this information and it's quite good to know that there are these services. It would probably be better if we receive more information about the services (GP-P1)

Some RACFs and RACF staff are unaware and preferring to still call GPs afterhours	• I apologise. I did not understand the concept fully well and I simply ticked no saying that I provide my own… If I can understand it a bit more, what does it involve? (GP-NP2)<br>• I don’t know whether the nursing home or the staff are aware of the services, because usually in the nursing home they’re good, they contact the GP first, but then what I find is in the middle of the night, they would fax me about what is happening to this resident, but how could I see that in the middle of the night or after 6 o’clock when I’m home. So at least I think if they know that there is this service – I don’t know whether they are aware (GP-P1)<br>• I don’t think they are using that much yet. Because I still get quite a few calls…at least once or twice on the weekend (GP-P5)<br>• facilities with higher staff turnover – so it's been also just – so re-engaging with the new staff, so we do offer regular refresh training sessions for the staff, just so that any new members of staff who come through are aware of the service (MED3)
Process of implementing program in RACFs<br>Challenges engaging GPs and RACF staff	• I think the major barrier initially was just getting the GP buy-in, and then, I suppose, the next barrier is just actually getting the facilities to use the service that is available to them (MED3)<br>• … it took the RNs a little while to sort of understand what that process is, and what it means. Obviously with COVID, it pushed us all into a situation of not wanting to send our people to hospital. So it took a little while for them just to grasp that this is the first call that they make to do that (RACF MG2)
GP reluctance to engage	• initially he [another practice doctor] said, "Look, I'm not going to do it. I'm really pissed off. Bugger paying them. They can pay me." (GP-P3)<br>• There were a number of GPs at each of the facilities who just point blank refused to sign any consent (MED 3)<br>• I provide my own afterhours care. And even during my, let’s say, holiday or time off, I still provide my own care (GP-NP1)<br>• After hours mainly on the phone, because, depending on the situation, whether they need to go to the hospital – we just give

	them advice like get the ambulance and send the patient to hospital straightaway. If it's something that is not urgent, it can be seen the next day, then I will go first thing in the morning the next day and see them. (GP-NP2)
Time pressure for RACF staff	• you need to speak to the nurse as the patient's communicator most of the time to tell us exactly and that, I think, for some nurses… they feel like it's a bit more time consuming. They need to spend time on the phone, looking through medications, talking to us, I think, they are feeling they can't deliver care to other residents. I think they really feel a sense of pressure and rush (MED2)
PHN support to set up program	• the program lead [from PHN] on `the pilot project, was just phenomenal, she actually drove out to some of the medical practices and had conversations with them and she also helped drive that initial getting all of the consents and the GP referral forms in (MED3)<br>• They provided the templates for our doctors to fill out, so the consent forms. They provided the spreadsheets to keep up-to-date residents' charts (RACF MG2)<br>• They were good. They were quite helpful. They always ask me if there are any problems. They can always come and talk to me about this problem. But so far, there's not much problem that I can see. (GP-P4)
MED is easy to set up and simple to use	• We didn't really have any challenges in setting up, it was nice and simple and easy really (RACF MG6-RN3)<br>• not just the registered staff in the ward, anybody can do it in the middle of the emergency situation. She can easily grab the – call her care staff to get the iPad and press the call, and then do the video calling for her (RACF MG3)
RACF staff need to adapt to technology (challenge)	• some previous staff, they're not really into technology. But, of course, with the assistance of our care managers or whoever's working with them, just assisting each other. So we manage to

**Training**<br>RACF Staff are educated and trained<br><br>RACF staff train other staff<br><br>Training refreshers and in service reminders provided and for new staff<br><br>Staff efficacy with telehealth - easily adapted to COVID telehealth consultations<br><br>RACF staff are also trained how to communicate with MED	sort that issue, and it's not really a big thing, but other than that, everything is okay. (RACF MG5-RN1)<br>• They [MED] provided the iPad education to the staff. We scheduled for online module education and handover. (RACF MG3)<br>• So they [MED] educated about it on how to use it [app]. And then, there’s the contact numbers as well, that we can contact in case that we need some follow up (RACF MG5-RN1)<br>• they provided us with the iPad and then they did training with me specifically and I then trained my deputies and my RNs. (RACF MG2)<br>• they [RACF] did it when we were rostered. So they do it, because not all nurses are on. So they do it daily. And then it was mentioned as well, in an RN meeting too. (RACF MG5-RN1)<br>• And we’ve done that again more recently because we’ve had new RNs starting and just to refresh all of us to make sure that we all remembered how to do it so (RACF MG6-RN3)<br>• The system we have is that there's a manager on-call - and so if the registered nurses are concerned about a resident and they’re having trouble getting on to the doctors [GPs] … we remind them about the after-hours telehealth doctor and say, “Why don't you give them a ring first before you ring an ambulance?” So that would jog their memories to do that. (RACF MG6-RN3)<br>• I feel like my team knows it better for when the COVID – was implemented, because they’re already used to the Telehealth After-Hours emergency calls. (RACF MG1)<br>• they would have had some training and would have had some expectations, I think, they use an ISBAR [Introduction, Situation, Background, Assessment, Recommendation] format, and so even when they speak, obviously they are time pressured, I spend the first three minutes just listening and absorbing. I’ve never had a consult without vital signs (MED2)

Infection control processes	• With the iPad, there is one supplied after hours, iPad, for the whole facility. But the staff are aware of the infection control practices, is to just wipe the iPad before and after use. (RACF MG3)
RACFs implement a process for escalating care that uses MED	• there is a consent form and there is a protocol in place where you have to call the GP up to 6 o'clock where you know if their GP is available, they come and review them but if they're not then you call My Emergency (RACF MG4)<br>• most of the local protocols would still be for the nursing staff to phone the local GP in the first instance, and when they are not available, to then approach My Emergency Doctor. (MED3)
**Consent**<br>Consent processes to participate in MED	• before we start anyone to be interviewed by an emergency doctor when they get admitted, we ask for consent from the families and also the GP signs off the consent as well (RACF MG3)<br>• we provided them with a draft letter or a template – which they could then amend as they would need for the facility, print on their aged care facility letterhead and arrange for signing with the patient or their next of kin (MED3)<br>• they asked me to sign the forms. Give all the information about my patients, who I have in the nursing homes. I had to supply them my – the family contact, family on the patient file. (GP-P5)<br>• getting consent from the GPs, that was our biggest challenge and the way that we tackled it was educating the GPs that we are really not taking their residents away from them but just making life more comfortable for residents as well as families and the hospitals by not sending residents to hospital when we can't get onto the GPs after 6 o'clock. (RACF MG4)
**Privacy**<br>Ensure resident's dignity and privacy of others	• you just have to make sure when people are recording a resident they are in dignified manner, the roommate is not being shown, it's only focussing on what the issue in relation to that resident that they're calling for (RACF MG3)
Privacy is balanced against need	• I think if I cannot see the patient, for example, a couple of weeks ago a facial swelling, and the video app wasn't working, so I asked the nurses, obviously this brings up a can of worms. Would you be

	able to send me a picture, and it brings up to another more ethical consideration about privacy. There needs to be balances of duty of care and emergent clinical need (MED2)
RACFs and MED have privacy policies in place	• [RACF] has developed a telehealth policy, that's only just come out a couple of months ago, around use of telehealth and confidentiality and stuff like that (RACG MG6-RN3)<br>• it's a confidential consultation, so when that occurs it's in a – I think they call it an encrypted space, I'm not entirely sure about the terminology there, and then also we adhere to privacy rules within the company. (MED3)
Privacy includes de-identifying reporting data	• also for de-identified data to be shared back at the end of the month via reporting to the PHN. (MED3)
**Communication processes**	• we got that in place, so everything is documented, the time that it was called, whatever ambulance has been called or after hour doctors have been called so that's all been logged in. (RACF MG4)<br>• we have a log for the telehealth that was provided to us by [PHN] and that gives you the date that it happened, what time, the resident's name, date of birth, age, gender, reason for presentation. It says the clinical impression or diagnosis, outcome as in do you need to call an ambulance, do you need to go to hospital, treat onsite? And whether the after-hours telehealth organised a script or pathology requests or anything like that, so that's the sort of paperwork that we fill out for telehealth. (RACF MG6-RN3)
Clarify guidelines (afterhours)	• So we just had to remind the staff to understand what time is after hours (RACF-MG3)
PHN engaging GPs	• when My Emergency was about to be initiated by PHN, all the GPs were also at our meetings, GPs meeting informed and asked and consulted. PHN really did above board (GP-NP1)<br>• We had an initial meeting about how to do it. That's about it. Maybe we might have had one or two communications from PHN about it. Very little actually. (GP-P3)

	• I think that GPs – I don't know whether it's just me or other GPs providing care have this information and it's quite good to know that there are these services. It would probably be better if we receive more information about the services. To be honest, I am not aware really of the extent of the services that can be offered to the residents (GP-P1).
Good communication is a priority for MED	• part of the program requirements is that we provide feedback to the client [NBMPHN] on a monthly basis and report against very specific key performance indicators… it would be the number of calls received per facility and then also confirm the number of calls that we managed in situ or the number of calls that were sent to an Emergency Department. (MED3)<br>• I always ask them, "Would you like it faxed or emailed?" they tell them and then I'll always remind the support officers of that, just so that that's done quickly. We've got a system where the senior clinical group, with My Emergency Doctor, will audit the paperwork, a discharge summary and all their notes, to make sure that it includes everything relevant and necessary (MED1)<br>• in all instances the [MED] administrative team will send back a clinical summary of the consultation and that will be via communication preference as stipulated by the facility, and they will also receive any imaging or pathology requests – that is sent directly to the facility. In some instances, a script is required that is our administrators will contact the pharmacy and fax through the script to the pharmacy to then fill. (MED3)
MED ensures reports go to GP	• we always send a clinical record or discharge summary to the aged care facilities, and a copy of that usually goes to the GP if we've got the GP's number, and often if I've had the chance to speak to the GP looking after them, I will ask for their fax numbers. It's not always readily available (MED2)
RACF staff forward communication and record in files	• after the call we get the report from the My Emergency Doctors, and we keep it for the next time GP comes, so that GP reviews what they did, the after-hours doctor has written (RACF MG3)

Communication with families and residents	• the paperwork is always there, and next time when I come, I always see there the paperwork generated, and the Telehealth doctors have write – some of them are quite detailed, some of them not so detailed. They put down the recommendation, what sort of treatment they have. (GP-P4)<br>• The carers, they're involved with the plan also. Any calls, any issues with residents, we call the family straight away. (RACF MG3)<br>• they're [instructions/treatment plans] discussed verbally with the family members and the resident (RACF MG6-RN3)<br>• For example, if they have had a minor head injury and they've been completely well and you did a risk assessment and you've had a discussion with the family and you say look, this is what we will do and you get them to repeat that. Verbal repetition is important. Writing it down on paper, the specifics and discussions [MED2)
Communicating advance care plans	• if it's really straightforward and the patient's unwell that's very straightforward, yes, they should go to hospital and then I always check, do they have an advanced care directive? Is that documented and what did the family think (MED1)<br>• we look at the advanced care plan and if it says palliative, not for hospitalisation, whatever, then we discuss that with the Emergency doctor, we talk to the family member as well, and the resident if the resident's able to talk (RACF MG6-RN3)
GPs provide health summaries and care plans which inform MED	• sometimes I do a comprehensive health summary. …The care plan is always there for the emergency doctor to review… (GP-P4)<br>• the Emergency doctor will have a discussion with the nurse regarding the resident and discuss any relevant information to that particular presentation. So if there is any information that – or a care plan that the GP may have in place, that it's then up to the nursing staff member, who would be familiar with that and who would have the actual resident's file in front of her or him, to discuss that with our specialist doctor (MED3)
Care Plans from MED implemented by RACFs and communicated to GPs	• the registered nurse would be liaising with the Emergency doctor and then putting a plan of care in place that the care staff would

	follow and follow-up with. If the care required isn't a medication or something, well, the RN would be responsible for making sure that that gets sent to the pharmacy to be dispatched first thing in the morning. (RACF MG6-RN3)<br>• any updates we automatically add to our care plans anyway. That's the normal process. (RACF MG2)<br>• So the nurse would just fax to me the assessment and the plan that has been made. (GP-P1)
**Experience of the MED Program**	
Variable GP engagement<br><br><br><br><br><br><br><br>***MED fills specific needs***<br><br>GPs willing to refer to MED when they're unavailable<br><br><br>24-hour care seen as benefit by all<br><br><br><br>MED extensively covers afterhours and acute care	• I would have liked to utilise it [MED] a bit more and see how it goes but one of our GPs who had quite a few residents refused to participate in the program and she said, "Nobody's seeing my residents except me." But the rest of the GPs were happy for another doctor to review their residents after-hours, they actually ticked a box that said only if I can't be contacted. So we still contact them first (RACF MG6-RN3)<br>• There were a number of GPs at each of the facilities who just point blank refused to sign any consent…I know for one of the facilities the GPs had actually changed their mind and agreed to sign consent for his residents (MED-3)<br>• if it's anything of emergency nature that needs urgent attention and I am unable to come, I always direct them to contact you [MED] (GP-P2)<br>• nursing home patients need 24-hour care. If there is a case where I am away at night-time, if they still can find someone if they have any problem and if there is any need of care, they can contact someone to review the patient, I'm happy. The patient is happy, the family are happy. (GP-P4)<br>• they're doing a good job because we use them for specific things. In the middle of the night a patient gets sick, which means the patient needs to be checked by a doctor. So it's good for that (GP-P3)

	• the GPs that comes here in the home, but at times, of course, it's very difficult. There's nothing; it's not as accessible as it is when we had the My Emergency Doctor (RACF MG5-RN1)
Care in the home is more available	• I've been really surprised at how much we are able to make a difference without the patient leaving their home and without us leaving our home. That's been, for me, a real surprise and makes it incredibly satisfying as a job. (MED1)
MED prescribing needs to be reviewed by GPs	• They [MED] might recommend that this patient should be on antibiotics, or this patient had a fall, has a lot of pain in the back, and they might write some analgesic or other drugs. They might write down "I prescribe Endone" for a few days or weeks. But the thing is, I don't know whether the change now can be written by the doctor who is actually prescribing in the Telehealth, because most of the time, I have to go back and write down what the doctor ordered, especially S8 drug. I usually don't like to write S8 myself unless I feel that it's necessary. I have to go and check because I didn't prescribe (GP-P4).
MED can be used for all populations	• it's [MED] a great app –very versatile for everyone. Anyone can use it. I hundred percent love it and support it because it's something that it can be used from toddlers right up to elderly and all culture and backgrounds because it is something that you can utilise straightaway (RACF MG4)<br>• different cultures backgrounds do benefit from it, even Aboriginals or the Chinese. (GP-P4)
RACF staff provide reassurance to residents	• we're all in collaboration with the care staff and emergency doctor. We give assurance that the residents are cared for, and their quality of life is met as much as we can. (RACF MG3)
MED can provide care in cross cultural situations and for non-verbal patients	• our specialist Emergency doctors are well trained in dealing with people from different cultural backgrounds and also different language, diverse language backgrounds…they'll always have the nursing staff available to translate if required – but we do have access to a translator service if needed (MED3)

	• For example, there's a lot of Filipinos working there, there are Filipino patients. The Filipino can interpret for them. There are other people – patients who are of Indian origin, there's an Indian there, there's a Chinese nurse there. So there are some nurses who speak their language that can interpret for them (GP-P4)<br>• If some people have any difficulty we will just use the non-verbal sign— we will tell the doctor, he can't speak (RACF RN2)
Sometimes MED used inappropriately	• We have been called for routine medications and that has created a bit of angst amongst us, but I see it is as if the patient or the resident does not have any other options, and for some reason, due to their aged care facilities, if it's inherent busyness or their time constraints are unable to get a GP to fill out the medication charts, and I will just say look, I will just do it. (MED2)
MED program is reliable and provides valued outcomes<br>MED prioritises need	• it's better than, for example in Nepean calls the aged care and the patient is really unwell, then the nurse will say, "This patient's really unwell" and they would get prioritised compared to, for example, a patient from Victoria who just needed a new prescription for their blood pressure medicine that they'd pick up the next day. (MED1)
MED contributes to ongoing care priorities	• I have to say that in the case of My Emergency Doctor, when they review a patient they direct your attention to what you need to review the patient because sometimes the patient may have a poly pharmacy and medication and they tell what you should do. So the general health care, the help is good because they direct your attention to what you need to do. (GP-P2)<br>• sometimes, you just need advice, even for chronic cases and then something changed, then sometimes just getting advice perspective from another doctor, I think, helps us give care or manage our residents. (GP-P1)
Ready access to care and advice	• that telehealth service is much faster to get my patients seen by a doctor afterhours. (GP-P4)

Nurses have rapid support	• look, it's good, it's good. It means I have after hours cover. So they're [RACF staff] much happier. I think they're comfortable they can contact someone after hours. (GP-P3)<br>• the top benefit is that we can provide care for them straightaway. I think that’s the best part of it, is that if we are in doubt, we can immediately call them, regardless of what the question is or what it is, they’re there to support and they will answer any questions that we have. That’s been a really big support. (RACF MG4)<br>• It meets my needs, because I get some advice, some support from other staff members in cases of emergencies which reduces the risk of neglect for the patient (GP-P2)<br>• GPs are confident that whatever recommendation from the telehealth, this is – it’s from their expert knowledge (RACF MG3)<br>• it has a very – great impact, …we can provide the immediate treatment that those residents that need. So it is less time for us our end as well because – we can just press the button, and wait for someone to pick it up… so it's very good, very helpful (RACF MG5-RN1)<br>• it’s basically given them another person to go to where the phone gets answered straightaway. So when you’ve got a resident who is unwell and there’s only so much we can do as registered nurses, and when you’re sitting and waiting for that doctor to get back to you, it is a little bit nerve wracking because you’re waiting to see, okay, what are we doing here? (RACF MG2)<br>• Before this was introduced, there were a few times where they [nurses] tried to book an after-hours GP to come for a resident, but it’s already 6 o’clock in the morning, there’s no-one who’s come… the after-hours GPs in the area are all fully booked. But with after-hours telehealth, just press the button and then they’re there. Easier access to meet their needs, and easier for them to be reviewed immediately (RACF MG3)
MED follow up with RACF staff provides reassurance	• when the GP has said, “I think the patient should go to ED”- the nurses have actually been really good when we reassure them, we

	say, “Look, we are emergency specialists and we will be sending you a letter” and what I have done a couple of times, where the nurse is very worried, I’ve actually called back in three or four hours to check how the patient’s doing and I’ve found that just that one or two-minute call back after that, they found really reassuring, and it’s often the patient has picked up. (MED1)
Video consultations are helpful	• it's great to have it there to know that we can ring somebody and they can actually visually see the resident after-hours if we need them, it's a great backup tool to have (RACF MG6-RN3)<br>• the benefit has been that the RNs know that there's something that’s just a phone call away, that they can actually speak to a doctor rather than talking over the phone. They can actually see the doctor and they can actually explain what’s going on and show the doctor the resident rather than just doing something by phone. So knowing there's a backup there if they need it. (RACF MG6-RN3)
In aged home service is faster than ED	• by the end of the day it saves time for the patient by having to wait for the emergency and gives them the service at the facility. (GP-P2)<br>• in the evenings and of a night and the weekend where it’s been very hard to get someone to come here and see them, and we’ve been able to care for our residents here rather than sending them to hospital for them get reviewed, ten minutes and then be sent back. So it has been a great help (RACF MG4)
Families appreciate fast access to care	• We regularly get feedback from families that they’re thankful that resident really needed to be sent to the hospital, or that patient needed to be seen by a doctor. And even if it’s a night time, they’re thankful that they are notified that their loved ones have been cared for, and that they’re being addressed and assessed in the middle of the night. (RACF MG3)<br>• feedback from the relatives or the guardians…from the nursing staff feedback, they always say, “Look, I’ve rung the guardian or the next of kin,” that this patient has been relieved by the after-hours Telehealth (GP-P4)

MED reduces ambulance transfers to ED and afterhours hospitalisation	• It’s certainly reduced the amount of ambulances that are coming here and the hospital presentations which benefits the residents at the end of the day. (RACF MG2)<br>• there’s a lot of falls in nursing homes and in the past, falls, they are always sent to hospital. But I feel that all this hospital admission or hospital consult in the emergency centre has reduced (GP-P4)<br>• it's great to know and gratifying to know that we do provide that extra level of support for them, because in the majority of cases, in 80% of the cases, we were able to help them avoid unnecessary transfer to ED. (MED3)
MED is cost effective	• It’s cost effective because it will save people going to hospital, use the resources or the ambulance because we know how expensive it is, and the hospital, stretching the facilities the emergency (GP-P2)<br>• from an efficiency point of view and a time management point of view, it’s certainly added to the aged care home. (RACF MG2)
Reduced stress and burden for GPs	• the GPs aren’t fielding a lot of calls, which I’m sure is giving them a better quality of life. (RACF MG2)<br>• I know when some of our doctors give us feedback too- having that My Emergency Doctor, we don't really have to message them, especially at night-time, during after-hours (RACF MG5-RN1)<br>• has relieved a little bit of my burden in answering calls. So, I get fewer calls from them now than other facilities (GP-P4)<br>• So now I get to sleep, and I get to have my weekends off (GP-P3)<br>• So I think it just lessens the stress from the GPs (GP-P1)<br>• sometimes I am away or I would be away on holiday, I couldn’t find any doctor to cover for me. So that was the advantage, I could go away for a few days and then I would say, look, I have no cover, so they could call the afterhours, that’s really very important (GP-P1)<br>• it saves my time because I don’t have to see everyone who is having an emergency, because when I have all the nursing homes, I can’t visit all of them. (GP-P2)

	• if I actually attended them, I bring in more money through Medicare. But I think quality of life is better for me anyway. So that means probably that the income is a bit less; it doesn't matter (GP-P4)
Reduced stress for residents when care managed in RACF	• its created a better quality of life for the residents- less discomfort, less waiting time, less anxiety that they feel that they're going to an unfamiliar environment or people that they're going to see. (RACF MG1)<br>• if the emergency doctor says, we have to keep the resident in the facility, just monitor them. If they deteriorate, that's the only time we send them to hospital. So that feeling where they stay in their home environment. They don't get stressed being transferred from one bed to the other stretcher, and then wait there for hours. That's one positive thing. (RACF MG3)<br>• When they go to hospital, particularly if they remain in ED all day, they come back distraught. They come back upset. It's an unsettling experience for them. And it's not necessary when you've got something like My Emergency Doctor (RACF MG2)
In home care reduces stress for families	• some residents, they don't really want to be transferred to the hospital. They want to stay here, which is good. Also, it lessens the anxiety of the family (RACF MG5-RN1)<br>• the resident was fairly comfortable, it was the family member, the daughter, that was really, really upset and emotional and demanding that he be seen right this minute and all this sort of stuff. So it was able to allay her fears knowing that she was actually there when the Emergency doctor was talking to her dad and reviewing him and she had it straight from the Emergency doctor that, no, he didn't need to go to hospital (RACF MG6-RN3)
Residents reassured by MED consultations	• We had a lot of positives. Just two nights ago, for example, we had a resident who was having a panic attack and wanting to go to hospital and she was determined that she was going to die if we didn't transfer her to hospital, but a call to My Emergency Doctors, she was able to settle and not needing to go. (RACF MG4)

	• they are reassured that they're not just left there overnight without no-one seeing them. So they get reassurance that they've been seen by a specialist doctor from emergency, that their conditions are okay. That they don't need to be sent to hospital (RACF MG3)
Good relationships between MED doctors and RACF staff	• there's a rapport you end up building with the aged care nurses. There are some that you wouldn't normally have much to do with, because they'd be in the facility and we'd be in the hospital and seeing how much work some of them are able to do out there in the community. I think that's a huge benefit (MED1)
MED learning how to provide care in the community	• one of the good things that have come out is it has improved my communication. It improves my emphasis of certain things. I need to think out of the box when I look at a patient, or how else can I provide care remotely. How else can I assist the client remotely with delivery of medications? We used to think the patient needs to go to hospital to get a medication, now we think out of the box. Can we get a pharmacy to deliver the medication on this hopefully it has assisted us in shifting our paradigm or delivering care in hospitals and delivering care onsite, and I think telehealth over any other vehicle has done that. (MED2)
**Communication from MED is efficient**	• we automatically get the results straightaway. Sometimes when they go to hospital, they come back with no discharge summary and things like that. So, they automatically send the report. We communicate this to the clinical team and then it gets followed up with whatever needs to be followed up and then filed. (RACF MG1)
MED can be suboptimal at times	• the RN mentioned that this patient [of another GP] is complaining he's got no vision. I said, "Since when?" So they said, "Maybe two days" …sometimes there is lack of communication maybe Saturday he had vision and after that he has no vision in his left eye and I said, "that's an emergency, I will ring the ophthalmologist and he will contact the family and ask them to take him straightaway." I think because that patient, maybe on the weekend, must have accessed this after-hours service. (GP-NP2)

MED offers team based care involving patients	• Generally, it would be a joint discussion where our Emergency doctor will have the discussion with the nursing staff member and the residents if possible. (MED3)
**Satisfaction of RACF staff**<br>MED supports an isolated workforce	• I found actually the nurses have been really happy – I personally felt a lot of positive feedback from the staff, and especially because it is out of hours and it must be quite isolating for the nurse. You know, they're often one nurse, to a whole nursing home. (MED1)<br>• general feedback via the facilities themselves seemed to be that they were quite happy with the service and just knowing that their residents are looked after during the after hours when the GPs are not available (MED3)<br>• So when you've got a resident who is unwell and there's only so much we can do as registered nurses, and when you're sitting and waiting for that doctor to get back to you, it is a little bit nerve wracking because you're waiting to see, okay, what are we doing here? What's the plan of attack? You've got a resident who is potentially quite upset or quite distraught and being able to just pick up the iPad and go straight through to an emergency doctor is just far more efficient for time as well, because nurses aren't waiting for those calls to make (RACF MG2)
RACF duty of care is supported	• They [RACF] get someone to depend on. Nursing home nowadays always have to document every single thing, because with the accreditation they want to make sure that the patient's safety is first. So if they can't get me, they can at least get the Telehealth doctor's advice and video facility, whether this patient needs to be in a hospital, whether it just be observation, 24 hours is sufficient. So in this case, the nursing home other staff are covered. (GP-P4)
Provides nurses with confidence	• So what it's done is allowed us to just give the RNs the confidence that you can monitor them like you would normally do in hospital and then go from there (RACF MG2)<br>• My Emergency will take away the clash or the friction between the nurse, the ambulance and the hospital because My Emergency Doctor will then be the third opinion… takes away the dilemma and

Complementarity with GP care<br><br>MED provides an incentive to attract more GPs to nursing home work	the hard feelings or the argument over the phone, which stress the RN more (GP-NP1)<br>• at the end of the day, you want to give them the care that they need…this is really important, I think that now…I would be accessing this service and I think it will be a very good complement to the care that we, as GPs, give to our residents. (GP-P1)<br>• We need to attract GPs to work in nursing homes and the after-hours service is actually an incentive because if you can say to GPs, "Well, you're not on call in the middle of the night, you're not on call 24/7 365 days a year," then it's much more attractive for GPs to work in nursing homes (GP-P3)
Use of other services	
RACFs prioritise MED over other afterhours services<br><br>Other services when needed<br><br>VACS and nurse practitioner services provide valued service within limited hours	• now our first call is to My Emergency Doctor. (RACF MG4)<br>• only we're calling on My Health [MED]. We didn't call other after-hour doctors. (RACF RN2)<br>• If ever they call the My Emergency Doctor it says to be followed up by Nepean Hospital VACS, then they come in here and they see that patient face to face (RACF MG3)<br>• I work hand in hand with them [VACS], and I know the people quite well. The nurses that come to the facility …often my patients might come from the hospital, and their service will … review the patient … after the patient has been discharged. (GP-P4)<br>• I think the nurse practitioners are better because they're embedded in the local community, they actually have knowledge of the community and they will often come in and physically see the patients as well. (GP-P3)<br>• I have the mobile phone of the VACS in my phone, I have consulted with them over the phone regarding my patients when I am hesitating about the treatment that the hospital can provide. I'm very well-aware that the VACS team is a specialist level and they don't work afterhours (GP-NP1)<br>• We have a nurse practitioner that we call upon but the hours that we use My Emergency Doctor is very different to the hours we can

Secondary triage helps avoid hospital<br><br>MED works with other services	utilise the nurse practitioner. She does come on the weekend or the afternoon but still doesn't cover all the hours that My Emergency Doctor covers, so both hand in hand are perfect (RACF MG4)<br>• The secondary triage when we used to call the ambulance they used to come but now actually if they think that's not an urgent thing they transfer us to them and they will determine whether we should actually call the ambulance or not so it's made it a little bit less transporting residents to hospital. (RACF MG4)<br>• we have a physio here on site, so if there's something that comes out of My Emergency Doctor, it gets referred to our physiotherapist straightaway, like any of our referrals would. Our medications can obviously be dealt with straightaway. So if My Emergency Doctor didn't have the capacity, for example, to change the meds, we would fax Med charts through to them. They'd change them and we'd deal with our pharmacy. (RACF MG2)<br>• we're utilising dieticians and we're doing pharmacy and all that stuff through Telehealth as well (RACF MG4)<br>• I entered the database this morning and I see that there is a local health district flying squad or a geriatric follow up or outreach service, so, we always emphasise that the ongoing care or the more complex management of a certain issue like, for example, changing medications or a more family meetings or another reassessment face to face would be through them, because they're better suited for it (MED2)
Improving Afterhours Care in RACFs and the MED Program	
Increase community funding to enhance nurse practitioner models of care	• A lot of money spent on a program just to help the hospitals when the actual thing we really need is more staff in the nursing homes. All that money could have been spent on some extra nurse practitioners (GP-P3)<br>• I think as an adjunct to telehealth whether it could involve some clinical nurses or clinical support staff from a network point of view to do a phone assessment of certain things as well. (MED2)

	• I think the whole COVID thing has made me realise that the most important thing for nursing homes is that they have a stable nursing workforce (GP-P3)
Need to promote MED as a complementary service and opportunity to work together	• we need to reframe so this is something new for them, new for the consumers and new for us, and we need to work together (MED2)<br>• I think at this time it's for all of us to be more aware that there is this service. I feel that, maybe there's a need for another series of information, just to reiterate or re-establish this again. (GP-P1)
Need to continue promoting MED in RACFs and address high staff turnover	• the main thing is just consistent level of communication - a few of the facilities with higher staff turnover – so re-engaging with the new staff so that any new members of staff who come through are aware of the service. (MED3)
Increase consultation between PHN and local GPs	• I think it's a good idea if we can have one of these meetings that we can give the program coordinator [NBMPHN] ideas, because we can share ideas, do a meeting through the Blue Mountain Division or webinar when we have time, we can talk together about how to - say in case of emergency, what do you want to do, Telehealth or do you want visit the facility (GP-P2)
Consider after hours funding for GPs<br>Sustainability needs to be considered	• It's important I think to fund afterhours consults with GPs (MED2)<br>• My Emergency has been financially subsidised by the PHN. At the end of this trial or pilot trial any aged care facility who would like to continue on, will have to pay themselves, and the cost is not cheap – any future decision about continuity with financial sustainability has to be considered well. (GP-NP1)
Importance of good internet connections	• I think government investment in funding for good Wi-Fi solutions. I think the technical and incentives for all aged care facilities to subscribe to at least one sort of telehealth provider and either through investment through a good 5G available or 4G available, having good Wi-Fi services is really, really important. (MED2)
Emergency doctors should have local knowledge	• I would love for [MED] doctors who know the locality and the area, it's very important. I trust their medical knowledge, but one thing I am a little bit reluctant or hesitant is that they don't have the knowledge of locality. (GP-NP1)

Increased collaboration across services needed	• would maybe be useful for us, for example, if we got a call on the Saturday maybe the virtual geriatrics team [VACS] could review the patient on the Monday morning because we've had some cases where the GPs don't seem to visit that often either for some nursing homes (MED1)<br>• I wish that sometimes they can be able to see the patient, but unfortunately it's not an option - if they can communicate with the Virtual After Hours, or they can send the nurse practitioner to come down and check the patient as well. (GP-P2)
Refining RACF communications with MED including through use of My Health Record	• the difficulty frequently…I would just love a system where they automatically took a photo of the drug chart and the past medical history and allergies. That's where I think there's a lot of difficulty, when the patient is on 20 different medications and you've got a relatively junior nurse trying to read them all out to us. And the past medical history, it's just very, very complex. That can be very time consuming… Even if they included the blood pressure and heart rate, that would just make it so much easier and then you could just focus on the actual presenting complaint. (MED1)<br>• I think if there are some facilities which have the My Health Record and if there's no opt out, the notes are on the My Health Record, that's useful for the next clinician who sees the patient, whether it's through the My Emergency Doctor or somewhere else to access the notes. (MED2)
Better access to advance care plans	• if they're already known to palliative care physician, to have that plan ready. And then also have an advanced care directive, if that could be provided (MED1)
Advance care directives save costs in afterhours care	• Our life would be made a million times easier if every nursing home resident had an advanced care directive. It would save so much pain to the poor old patient, and of course save the country millions, hundreds of millions. (MED 1)
Increase Telehealth to include palliative care	• I personally think palliative care via video consult with someone who is pretty sick or they are expected to pass away, I think there is value in us trying to save them to go to hospital. (MED2)

Extend telehealth service to normal hours with GPs<br><br>Extend service beyond nursing homes to private residents and with disabilities<br><br>Continue MED afterhours service	• It would be so good if it was available throughout the day and we had our GPs on board to do that, then we could make a call through to them without having to present at ED, instead of waiting and chasing GPs to get things done and residents looked at, I think for me it'd certainly reduce day admissions. (RACF MG2)<br>• so many senior people at Springwood at homes this would be a good service. This would be the expectation if it could extend to that. And people with disability at home of course. (GP-P2)<br>• it would be good if we continue on with the service, for long as the service is available to us. Because it's really good that there is something that's readily available to assess or see the residents in times of emergency after hours (RACF MG3)
